# Thermo-Structural Characterization of Phase Transitions in Amorphous Griseofulvin: From Sub-T_g_ Relaxation and Crystal Growth to High-Temperature Decomposition

**DOI:** 10.3390/molecules29071516

**Published:** 2024-03-28

**Authors:** Roman Svoboda, Kateřina Kozlová

**Affiliations:** Department of Physical Chemistry, Faculty of Chemical Technology, University of Pardubice, Studentská 573, 532 10 Pardubice, Czech Republic; katerina.kozlova1@student.upce.cz

**Keywords:** amorphous griseofulvin, DSC, crystal growth, structural relaxation, particle size

## Abstract

The processes of structural relaxation, crystal growth, and thermal decomposition were studied for amorphous griseofulvin (GSF) by means of thermo-analytical, microscopic, spectroscopic, and diffraction techniques. The activation energy of ~395 kJ·mol^−1^ can be attributed to the structural relaxation motions described in terms of the Tool–Narayanaswamy–Moynihan model. Whereas the bulk amorphous GSF is very stable, the presence of mechanical defects and micro-cracks results in partial crystallization initiated by the transition from the glassy to the under-cooled liquid state (at ~80 °C). A key aspect of this crystal growth mode is the presence of a sufficiently nucleated vicinity of the disrupted amorphous phase; the crystal growth itself is a rate-determining step. The main macroscopic (calorimetrically observed) crystallization process occurs in amorphous GSF at 115–135 °C. In both cases, the common polymorph I is dominantly formed. Whereas the macroscopic crystallization of coarse GSF powder exhibits similar activation energy (~235 kJ·mol^−1^) as that of microscopically observed growth in bulk material, the activation energy of the fine GSF powder macroscopic crystallization gradually changes (as temperature and/or heating rate increase) from the activation energy of microscopic surface growth (~105 kJ·mol^−1^) to that observed for the growth in bulk GSF. The macroscopic crystal growth kinetics can be accurately described in terms of the complex mechanism, utilizing two independent autocatalytic Šesták–Berggren processes. Thermal decomposition of GSF proceeds identically in N_2_ and in air atmospheres with the activation energy of ~105 kJ·mol^−1^. The coincidence of the GSF melting temperature and the onset of decomposition (both at 200 °C) indicates that evaporation may initiate or compete with the decomposition process.

## 1. Introduction

**Scheme 1:**
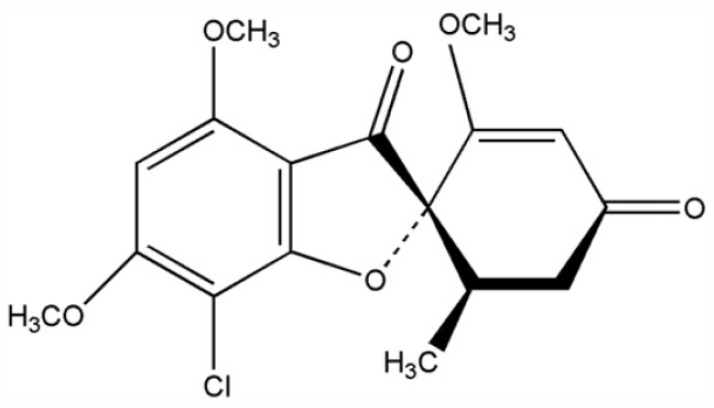
Chemical structure of griseofulvin.

Griseofulvin (GSF; see Scheme 1 for its chemical structure), a naturally occurring antifungal agent, has garnered attention for its diverse pharmacological activities, including anticancer and antiviral properties [1]. It is commonly used in the systemic treatment of dermatophyte infections, such as tinea cruris, that do not resolve after topical therapy [2]. Griseofulvin is known for its poor water solubility and is typically administered orally to treat topical fungal infections of the skin and hair [3]. Recent studies have also explored its potential as an apoptosis-inducing agent in the treatment of lymphoma and multiple myeloma [4]. Furthermore, GSF has been investigated for its potential to induce mitotic arrest and inhibit the hedgehog signaling pathway [5]. The drug has also been found to impair the intraerythrocytic growth of Plasmodium falciparum through ferrochelatase inhibition, although it lacks activity in an experimental human infection study [6]. Additionally, GSF has been reported to induce the depletion of heme, making it a well-known inducer of acute porphyria-attacking substances [7].

In terms of drug delivery, various methods have been explored to study and enhance the bioavailability of GSF [8,9,10,11]. For instance, the use of supercritical fluid technology has been employed to increase the dissolution rate and bioavailability of GSF [12]. Nanoparticle-based drug formulations have also been developed to improve the in vitro biocompatibility of GSF against human keratinocyte cells [13]. Furthermore, the synthesis of polymer-coated GSF nanoparticles has been investigated to enhance its bioavailability through sustained-release preparations [14]. These studies highlight the ongoing efforts to improve the delivery and efficacy of GSF.

In an alternative research path, GSF has been the subject of bioavailability testing in the form of amorphous solid dispersions (ASDs). Studies have shown that GSF ASDs prepared using specific methods did not exhibit crystallinity or increased impurity, even after storage under harsh conditions, indicating the potential for improved stability and efficacy [15]. In its pure amorphous form, GSF has been reported to exhibit peculiar crystallization behavior, with nucleation being imitated (or massively enhanced) by the quench-induced cracks in otherwise smooth amorphous surfaces [16]. Moreover, for intensely milled GSF (either amorphous or crystalline), fast crystallization occurs with the onset of the glass transition or even slightly below the glass transition temperature (*T_g_*) [17]. This behavior may be a consequence of GSF exhibiting a so-called diffusion-less glass-crystal (GC) growth, i.e., rapid formation of the crystalline phase along the micro-cracks below *T_g_* [18,19]. The rapid sub-*T_g_* crystal growth in GSF is associated with extremely fast surface diffusion (outpacing bulk diffusion by a factor of 10^8^), where the key role is played by the GSF polar functional groups not participating in the intermolecular hydrogen bonding [20,21]. 

In modern pharmaceutical research and industry, the high-temperature stability of amorphous drugs becomes increasingly more relevant due to individual patient targeting via controlled-release tablets and capsules, where the key processing technology is either hot-melt extrusion (HME) or 3D-printing (3DP) [22,23,24,25]. Since GSF is a high-dosage drug [9], processing temperatures in the vicinity or even exceeding its *T_g_* need to be used as a bare minimum in the corresponding formulations in order to achieve the required cohesion of the HME/3DP-produced filaments [26,27,28,29,30,31,32,33]. This makes the temperature-induced crystallization of GSF a very relevant issue, even at temperatures close to *T_g_*. Despite the decade of relatively intense research on the microscopic crystal growth behavior, no systematic quantifiable characterization of the macroscopic crystallization kinetics of amorphous GSF has been published to date. In the present paper, full thermo-kinetic characterization of as-prepared amorphous GSF will be performed, including the kinetics of the structural relaxation, cold crystallization, and thermal decomposition processes. Mutual correlations between these phenomena (including the literature data on microscopically observed crystal growth) will be sought and discussed. Particular attention will be paid to the fast crystal growth and nucleation in the glass transition range. In addition, the kinetics data obtained by means of thermo-analytical methods will be interpreted in accordance with the structural analysis provided by spectroscopic and diffraction methods.

## 2. Results

**Figure 1:**
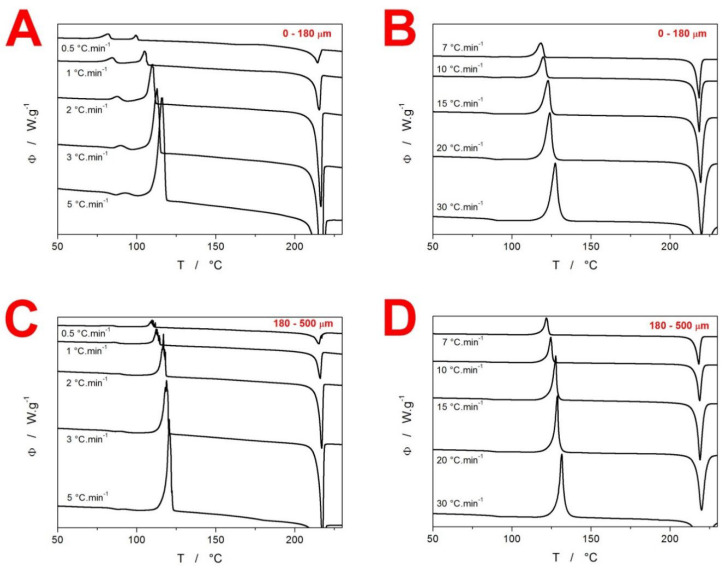
(**A**–**D**) DSC curves obtained for the two GSF particle-size-differentiated powders at different q^+^. Exothermic effects evolve in the upward direction.

The base calorimetric characterization of the prepared GSF powders is shown in Figure 1 (the zoomed-in and un-zoomed versions of the figures are included in the Supplementary Materials). The phenomena characteristic for the heating of an amorphous material can be identified on the majority of displayed DSC curves. In the ~75–90 °C temperature range, the endothermic step-like change is located, indicating the glass transition. Note that the glass transition signal is present under the majority of experimental conditions (it is just relatively weak in comparison to the other displayed signals and thus hardly visible); the only exceptions are the very low q^+^ (0.5–2 °C·min^−1^) and 0–180 µm, where the exothermic peak fully overlaps with the glass transition. However, since even under these conditions a second exothermic occurs at a higher temperature, it is clear that at that moment a certain portion of the amorphous phase has to be still present in the sample and, hence, there is always an underlying glass transition effect below the dominating exothermic signal (later defined as pre-crystallization peak). Further, a sharp exothermic peak occurs between 95 and 140 °C (depending on the applied q^+^), which corresponds to the main amorphous-to-crystalline transformation in GSF. An interesting feature associated with the main crystallization peak is its smoothness, which largely deteriorates for the coarse GSF powder (180–500 µm) at low q^+^. This is a consequence of the GSF crystallizing primarily from the surface and mechanically induced defects (which is characteristic for low-molecular organic glasses [34,35,36]), and in the case of only several large powder grains forming the sample mass, the growth of each individual crystal (which there are not many of) manifests as an individual exothermic sub-peak. The mutual shifts of these crystallization sub-peaks along the T axis are then caused by the variety of the grain sizes and also by energy barriers associated with the damage/defects of each individual grain surface. On the other hand, in the case of high q^+^, the energy surplus delivered by the rapid heating to higher T erases the difference between the activation energy barriers of individual active nucleation/growth centers. Contrary to the coarse GSF powder, the crystallization of the fine grains (with a high density of mechanically induced defects) manifests through smooth exothermic peaks due to the very large number of rather small crystallites (with sterically constrained dimensions, determined by the mutual contact) forming in the process. The signal envelope of the correspondingly high number of overlapping crystallization sub-peaks is further smoothened by a constant energy delivery via the heat developed during the exothermic amorphous-to-crystalline transformation. As a last observed effect on the DSC curves, a relatively sharp endothermic peak with onset at ~200 °C and extrapolated onset at ~215 °C denotes the melting of the crystalline phase.

Apart from these (rather generic) phenomena, the main variable outcome for the matrix of q^+^ and particle size conditions is the occasional occurrence of an additional exothermic crystallization peak, very closely following the glass transition effect. From now on, this effect will be denoted as the “crystallization pre-peak”. For both powder sizes, the crystallization pre-peak is detectable up to q^+^ = 10 °C·min^−1^ but significantly decreases in magnitude with q^+^. Whereas for the 180–500 µm powder, the crystallization pre-peak is still rather negligible even at 0.5 °C·min^−1^, for the 0–180 µm powder, the magnitude of the crystallization pre-peak is comparable to that of the main crystallization peak at 1 °C·min^−1^, and even exceeds the main crystallization peak in magnitude at 0.5 °C·min^−1^ (exact comparison of the crystallization enthalpies will be given in Section 3.2). This clearly indicates that this rapid *T_g_*-induced crystallization originates from the mechanically induced defects (as results from the comparison of the two particle size fractions) and that either the corresponding crystal growth is very slow or that the nucleation of this phase proceeds slowly in a rather narrow temperature interval. As the pre-peak crystallization proceeds only with the onset of *T_g_*, it is not consistent with the concept of glass-crystal growth [35,36,37] (which is essentially restricted to the sub-*T_g_* temperatures). Similarly, surface diffusion (as a more general source of growth rapidity) is largely increased below *T_g_* but loses its importance above *T_g_*, where viscosity is the dominant growth-driving force [20,35,37]. Accordingly, it is the nucleation that appears to be responsible for the strong q^+^-dependence of the crystallization pre-peak evolution (further evidence will be given in the following paragraphs that will introduce the structural relaxation experiments). As such, the number of nuclei formed on the mechanical defects [16,36,37] determines the consequent amount of the crystalline phase formed during the crystallization pre-peak. The internal consistency of this hypothesis is in agreement with the development of the crystallization pre-peak in dependence on both q^+^ and powder particle size. 

Compared to some recently published and similarly characterized amorphous drugs, GSF shows a rather low thermal stability (similar to nimesulide NIM [38] and unlike indomethacin IMC [39]), which can be quantified in terms of the Hruby criterion (see Equation (1)) as *K_H_* ≈ 0.3 (considering the main crystallization peak), and *K_H_* close to 0 for the crystallization pre-peak.

(1) $$K_{H}=\left(T_{c}-T_{g}\right)/\left(T_{m}-T_{c}\right)$$

where *T_g_*, *T_c_*, and *T_m_* are the glass transition, crystallization, and melting temperatures, respectively. Note that the typical values of *K_H_* range between 0.5 (below-average glass-formers) and 1.5 (above-average glass-formers) [40]. The values of *K_H_* ≤ 0.3 are even obtained for the materials that cannot be prepared in bulk but only by means of a physical vapor deposition technique [41]. This indicates that, first, there can be a large disproportion between the glass stability and glass-forming ability, and, second, that even the bulk GSF amorphous matrix needs to be considered a low-stability material, for which knowledge of crystallization kinetics is highly relevant for processing purposes.

Similarly to nimesulide (and unlike indomethacin), no apparent polymorphic changes or alternatives were revealed with q^+^ or particle size, which means that GSF has only one stable low-T polymorph. Note that the majority of known GSF polymorphs form from solution [42]. The widened onset and endset parts of the melting peak measured at higher q^+^ can be attributed to the larger variety of crystallite sizes (which is in correspondence with a broad range of powder size distributions)—this is in contrast to the results measured for narrow size distributions in [38,43]. Also, GSF is the only drug, out of the three discussed, that exhibited *T_g_*-induced crystallization, which may be associated with GSF having one of the fastest surface diffusions (compared to bulk diffusion at *T_g_*) [20]—more on this in the next paragraphs. Quantification of the base thermo-kinetic behavior depicted in Figure 1, i.e., the values of characteristic temperatures and enthalpies associated with the depicted phenomena, is introduced in the Supplementary Materials: the *T_g_* data indicate that at q^+^ < 2 °C·min^−1^, the crystallization pre-peak completely overlaps with the glass transition signal of the finely powdered GSF; the crystallization pre-peak occurs for q^+^ < 10 °C·min^−1^ similarly for both GSF powders, which indicates that a relatively low number of mechanical defects is actually needed for the nucleation to take place, and that the nucleation time is indeed the most important factor here.

**Figure 2:**
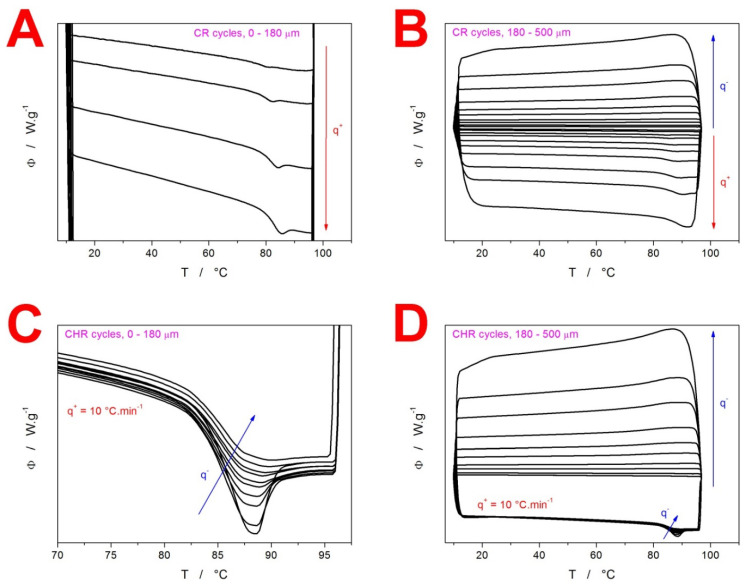
DSC records of the CR and CHR cycles measured for the amorphous GSF. The arrows and symbols q^−^ and q^+^ denote the parts of the DSC data in which the cooling and heating steps, respectively, of the given cycles are shown. Absolute magnitudes of q^−^ and q^+^ applied in the corresponding steps of the cyclic program increase in the directions of the given arrows. Exothermic effects evolve in the upwards direction. (**A**) Set of CR cycles measured for the 0–180 µm powder and zoomed-in on the heating scans performed at four lowest q^+^ (0.5, 1, 2, and 3 °C·min^−1^). (**B**) Full set of CR cycles measured for the 180–500 µm powder. The upper half of the curves corresponds to the cooling steps; the lower part of the curves corresponds to the heating steps (the farther from the center, the higher the absolute rate value). (**C**) Set of CHR cycles measured for the 0–180 µm powder and zoomed-in on the heating scans at 10 °C·min^−1^ in the glass transition range. The larger the relaxation peak, the lower the q^+^ of the preceding cooling step. (**D**) Full set of CHR cycles measured for the 180–500 µm powder. The upper half of the curves corresponds to the cooling steps (the farther from the center, the higher the cooling rate); the lower part of the curves corresponds to the heating steps.

In addition to the base characterization and crystallization kinetics, the DSC was also used to perform the structural relaxation experiments—the CR and CHR cycles (see Figure 2). As the structural relaxation in low-molecular organic glasses proceeds on a molecular level [44,45,46], it is not affected by the amorphous grain size. The DSC records of the CR and CHR relaxation cycles were indeed identical for both GSF powder batches. 

Therefore, the data obtained for the 0–180 µm powder are specifically displayed zoomed-in on the heating scans, which will be used in Section 3.1 for the evaluation of the structural relaxation kinetics. The most important aspect of these data is that it was possible to measure them at all. Judging from the heating scans displayed in Figure 1A, intense crystallization should occur during the cycles performed at low q^+^, which would degrade the amorphous nature of the sample. This is, however, evidently not the case. No change in the amorphous character of the samples is also evidenced by the constant Δ*c_p_* value (corresponding to the difference of the glass and undercooled liquid heat capacities; see Figure 2C). Note that the shift of the DSC signal with the progression of individual CHR cycles is caused by sintering of the amorphous grains, as confirmed visually upon opening the DSC pans after the measurements. The key reason for the suppression of the crystal growth appears to be the fast heating to the temperature above *T_g_* preceding the first cycle (initial equilibration of the amorphous material in the undercooled liquid region). During this heating, the mechanical defects acting as the nucleation centers seem to perish due to the viscous-flow-evoked self-healing of the major portion of the micro-cracks and other akin defects. This observation represents the above-advertised validation of the hypothesis regarding the origin of the crystallization pre-peak. Sole nucleation, regular above-*T_g_* crystal growth, or sub-*T_g_* GC growth would all manifest extensively during the CR and/or CHR relaxation measurements. It is only the unique combination of the nuclei formed specifically on the mechanical defects that is affected by the initial above-*T_g_* annealing, and that is thus behind the formation of the crystallization pre-peak. Apart from the anomalous annealing–nucleation–crystallization interaction, the GSF structural relaxation DSC data exhibit somewhat smaller relaxation overshoot (endothermic peak in the glass transition region) [47,48] compared to the IMC [39] or NIM [38], which testifies to the larger structural diversity and associated variability of the relaxation movements (more on this topic in Section 3.1).

**Figure 3:**
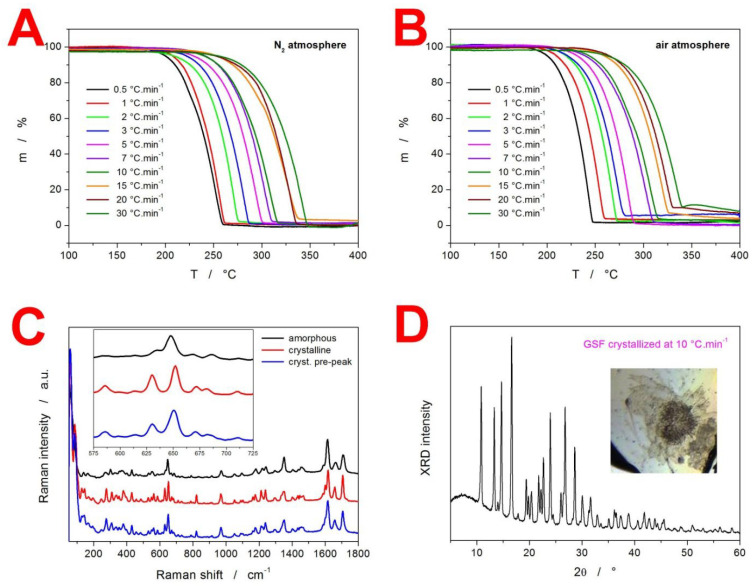
(**A**) Set of TGA curves obtained for GSF under N_2_ atmosphere. (**B**) Set of TGA curves obtained for GSF under air atmosphere. (**C**) Raman spectra obtained for the amorphous, fully DCS-crystallized and partially crystallized (to the crystallization pre-peak) GSF. The inset shows the spectra zoomed-in on the 575–725 cm^−1^ region. (**D**) XRD pattern obtained for the fully DSC-crystallized GSF. The inset shows an optical micrograph depicting a GSF crystal grown at 75 °C from a micro-crack artificially made on a smooth bulk surface of an amorphous GSF droplet. Dimensions of the micrograph are ~0.5 × 0.5 mm.

The high-T thermal degradation of GSF was explored by means of thermogravimetry—the corresponding mass loss curves are depicted in Figure 3A (N_2_ atmosphere) and in Figure 3B (air atmosphere). Rather similar onset temperatures for the decomposition process and the q^+^-induced spans of these onsets observed for the two reaction atmospheres confirm that the potential oxidation does not significantly (from the practical point of view held by pharmaceutic research) affect the GSF decomposition. The oxidative atmosphere leads to incomplete gasification at high q^+^ (char-like residuum was identified in the Al pans; the residuum masses varied between 5 and 10%). An akin decomposition process was also observed for the IMC and NIM [38,39]. In addition to the thermoanalytical techniques, Raman spectroscopy and XRD were used to characterize the amorphous-to-crystalline transformation of GSF—see Figure 3C,D. Both characterization techniques have clearly identified the polymorphic form I (based on the comparison with the literature data [40,49,50]) as a practically exclusive product of the crystallization from the glassy state. The following attribution of the Raman bands was suggested [50] for GSF: 0–200 cm^−1^ translational and vibrational lattice modes; 350–460 cm^−1^ and 590–690 cm^−1^ C-Cl vibration; 823 cm^−1^ cyclohexene ring; 968 and 1659 cm^−1^ benzene ring; 1150–1200 cm^−1^ C-O-C vibrations; 1200–1420 cm^−1^ CH, CH_2_, and CH_3_ deformations; 1570–1635 and 1585–1735 cm^−1^ C=O vibrations. As one of the largest differences between the amorphous and crystalline GSF Raman spectra occurs in the 600–700 cm^−1^ spectral range (attributed to the C-Cl vibration), this region was chosen to assess the nature of the crystallization pre-peak. A 0–180 µm powder sample was heated at 0.5 °C·min^−1^ to 82 °C and a Raman mapping was performed on the surface of several grains. Whereas a majority of the measured Raman spectra were very reminiscent of the form I crystalline signal, the bands were still not fully developed, indicating an overlap of the amorphous and crystalline signals—see the blue spectrum in Figure 3C. Moreover, approx. 40% of the spectra still indicated fully amorphous regions on the surface of the grains. Since the temperature of 82 °C corresponds to the maximum of the crystallization pre-peak for those measurement conditions, one would certainly expect the whole grain surface to be covered by the crystalline phase if the nucleation/growth were strictly surface-located. The significant presence of the amorphous phase on the grain surface unambiguously indicates that the nucleation and the consequent growth occur along the micro-cracks and stress zones located throughout the whole grain volume and that the crystallization pre-peak is not associated with preferential surface formation of the crystalline phase. 

The XRD pattern shown in Figure 3D was obtained for a fully DSC-crystallized GSF (0–180 µm powder; 10 °C·min^−1^ to 120 °C)—no sign of any polymorph other than the dominant form I occurs. This again indicates that only the GSF polymorph I forms during the dominant crystallization peak (occurring above the *T_g_* range and accounting for the whole material’s volume), because under these conditions, the main peak represents the absolute majority of the formed crystalline phase—see Figure 1B. The inset in Figure 3D shows a scratched GSF droplet solidified on a microscopic slide that was crystallized in a drying oven at 75 °C. The central dark mass represents the primary crystallite formed from the damaged surface; the light-brown shadow spread over the underlying pale-blue background (amorphous phase) represents the thin surface layer of the crystalline phase. The sharp and long edges of the surface crystalline phase indicate that the growth propagates along (and is determined by) the micro-cracks formed during the scratch. Note that the black parts of the micrograph only correspond to the contrasted part of the sample, where a deep fracture of the originally intact droplet occurs.

## 3. Discussion

Based on the thermo-analytical (DSC and STA) data introduced in Section 2, the kinetics of the observed phenomena will be described in terms of the current state-of-the-art solid-state models. The section will be split into sub-sections corresponding to each of the studied processes (structural relaxation, crystal growth, and thermal decomposition). In addition, Section 3.4 will be added, discussing the mutual relationships between these phenomena.

### 3.1. Kinetics of Structural Relaxation in Amorphous GSF

As was shown in Figure 2, the structural relaxation process manifests itself very similarly for both particle-size-differentiated powders. This finding will be utilized to evaluate the reproducibility of both the instrumental performance and material homogeneity. The nowadays standard mathematical framework for the description of the structural relaxation kinetics in the glass transition range is the phenomenological Tool–Narayanaswamy–Moynihan (TNM) model [51,52,53]:

(2) $$\Phi (t)=\frac{dT_{f}}{dT}=\frac{c_{p}-c_{pg}}{c_{pl}-c_{pg}}$$

(3) $$\Phi (t)=\exp\left[-{\left(\int_{0}^{t}\frac{dt}{\tau \left(T,T_{f}\right)}\right)}^{\beta }\right]$$

(4) $$\tau (T,T_{f})=A_{TNM}\cdot \exp\left[x\frac{\Delta h^{*}}{RT}+(1-x)\frac{\Delta h^{*}}{RT_{f}}\right]$$

where *Φ*(*t*) is the normalized DSC signal from the glass transition range, and *c_p_*, *c_pg_*, and *c_pl_* are the measured, the extrapolated glass heat capacity, and the extrapolated undercooled liquid heat capacity, respectively. Furthermore, *t* is time, *τ* is the relaxation time, *β* is the non-exponentiality parameter, *A_TNM_* is the pre-exponential factor, *x* is the non-linearity parameter, ∆h* is the relaxation activation energy, *R* is the universal gas constant, *T* is temperature, and *T_f_* is the fictive temperature. The enumeration of Equations (2)–(4) can be done by a variety of methods [54]. Recently, the combination of the ∆h* + *A_TNM_* evaluation [55,56] from the CR cycles and the evaluation of *β* + *x* from the simulation-comparative method [57] applied to the CHR cycles was shown to be very reliable and simple to use. Note that another benefit of this method is that it is not as prone to the data-distortive effects as, e.g., the curve-fitting approach based on non-linear optimization [58]. Starting with the determination of ∆h*, the equation (Equation (5)) utilizing the shift of the maximum of the relaxation peak T_p_ with q^+^ during the CR cycles (Figure 2A,B) is paired with the correction factor (Equation (6)), where ∆h*_true_ is the correct value of the activation energy, and ∆h*_exp_ is the activation energy determined using Equation (5). Note that the constants in Equation (6) are a result of a fit of the experimental data described in [56]. As a whole, Equation (6) functions as a correction factor for the conversion between the evaluated and true values of the activation energy.

(5) $$-\frac{\Delta h_{\exp}^{*}}{R}={\left[\frac{d\ln\left|q^{-}\right|}{d\left(1/T_{p}\right)}\right]}_{q^{-}/q^{+}=const.}$$

(6) $$\frac{\left(\Delta h_{\exp}^{*}-\Delta h_{true}^{*}\right)\times 100\%}{\Delta h_{true}^{*}}=4.218\cdot 10^{-5}{\left(\frac{\Delta h_{true}^{*}}{R}\right)}^{2}+4.841\cdot 10^{-2}\left(\frac{\Delta h_{true}^{*}}{R}\right)+\left[9.885\cdot 10^{1}/\left(\left(\frac{\Delta h_{true}^{*}}{R}\right)-1.276\right)\right]$$

**Figure 4:**
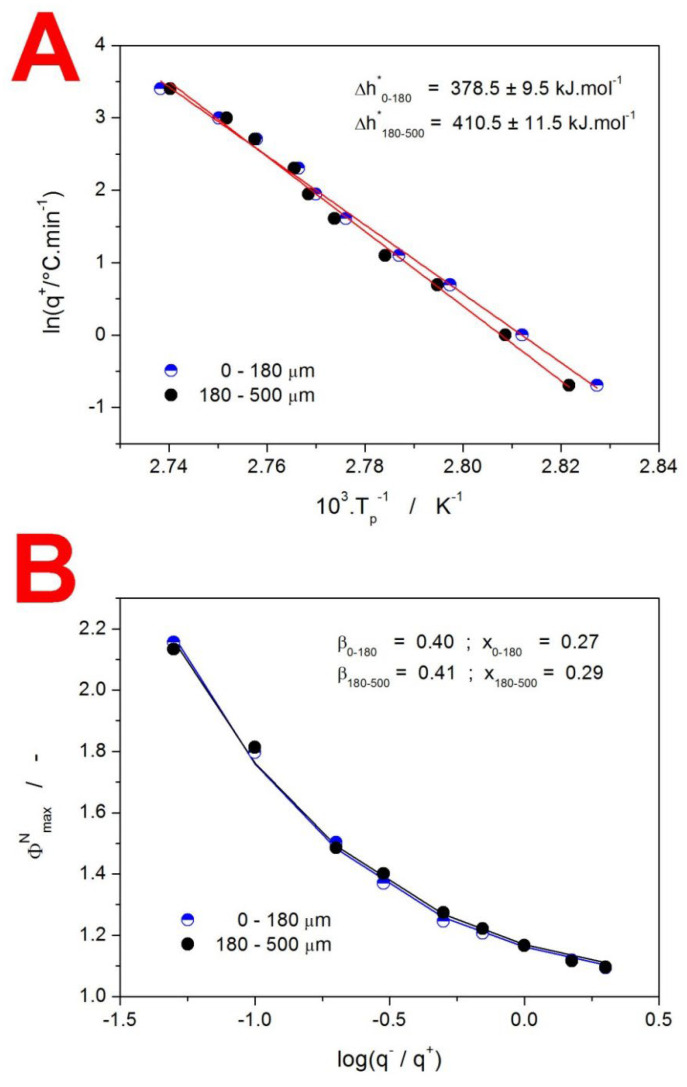
(**A**) Evaluation of Δh* from CR cycles according to Equations (5) and (6). (**B**) Evaluation of *β* and *x* by means of simulation-comparative method. Points represent the experimental data; curves in matching color indicate the best corresponding simulated *Φ^N^_max_*-log(*q^−^*/*q^+^*) dependence.

The evaluation of the present CR cycles data is depicted in Figure 4A. Both dependencies are fairly linear, as indicated by the relatively small errors of the determined ∆h* values (the listed activation energies are already corrected via Equation (6), which rules out any major instrumental data-distortive effects). 

Nonetheless (contrary to the initial assumption), there is a significant difference between the two dependences obtained for the present GSF powder fractions. Namely, the coarser powder exhibits *T_g_* (and, by extension, also *T_p_*) at slightly higher temperatures during the low-q^+^ scans. Since this deviation happens at low q^+^ (rather than high ones), it cannot be associated with any kind of thermal gradient (within the sample or on the path from the sample to the DSC sensor). As such, the possibility of the structural relaxation process being affected by the particle size needs to be considered. Firstly, the influence of the potential presence of small amounts of the crystalline phase (not recognizable from the DSC data) can be substantiated based on the higher tendency towards crystal growth in the case of low q^+^ and the high density of defects (0–180 µm powder). The apparent decrease in *T_g_* or *T_p_* would, however, not be consistent with the recent observations for other partially crystalline materials (including amorphous IMC) [38,39], where even high crystalline content did not affect the position of the glass transition—bear in mind that for the present GSF data, only a very small number of crystallites might theoretically escape the attention. The second reason for the observed decrease in the *T_g_* of the fine GSF powder may be associated with internal stresses induced by the mechanical processing (grinding). Contrary to the potential crystallinity being strictly located along the micro-cracks and acting rather as an inert phase, the internal stress would be distributed throughout the material volume, (potentially) affecting it on a molecular level. Such an explanation may be plausible considering the anomalously high surface diffusivity of GSF (and the associated lack of intermolecular bonding), which could distinguish the material from other low-molecular organic glasses. 

With the knowledge of ∆h*, the pre-exponential factor *A*_TNM_ can be determined via curve-fitting [58]: ln (*A*_TNM_/s) = −125.5 (0–180 µm) and −136.2 (180–500 µm). While A_TNM_ only indicates the position of *T_g_* on the T axis and, as such, its determination cannot be significantly influenced by the data-distortive effects, the evaluation of *β* and *x* (responsible for the shape of the relaxation peak) is, on the other hand, faulted by any slight data distortion. It has thus been found a better approach to employ the simulation-comparative method [57], which does not rely on the course of the DSC signal throughout the whole glass transition range (as in the curve-fitting) but instead evaluates only the more robust maximum height of the relaxation overshoot *Φ^N^_max_*. In particular, the *Φ^N^_max_* quantity is evaluated for a series of CHR cycle heating scans, and the data are plotted in dependence on log(*q^−^*/*q^+^*). Similar dependences are then evaluated for CHR data series simulated for different combinations of TNM parameters *β* and *x* (with known ∆h^*^ and *A*_TNM_); the best match then corresponds to the sought *β* and *x* values. In the present case, the following strategy was adopted for faster mapping of the *Φ^N^_max_*-*β*-*x* hyperspace. In the first round, the simulations were performed with the 0.1 resolution in the full range (0–1) of both TNM parameters; consequently, the resolution was increased step-wise to 0.05, 0.02, and 0.01, with the explored range of the hyperspace being halved and centered around the previous best guess with each new round. The resulting experimental and optimized *Φ^N^_max_*- log(*q^−^*/*q^+^*) dependences are shown in Figure 4B, together with the corresponding *β* and *x* parameters. The *β* and *x* values determined for GSF are very similar to those obtained for a finely powdered amorphous IMC [39]. Interestingly, whereas the amorphous IMC exhibited a significant dependence of parameters *β* and *x* on particle size, practically identical *β* and *x* values were obtained for both GSF powders. Regarding the absolute values of the TNM parameters, GSF ones lie in between the values obtained for the two IMC powders, so it is not conclusive whether the potential deviations should be sought for very finely milled GSF or for the amorphous bulk. Compared to the structural relaxation in amorphous NIM, both GSF shape-responsible parameters are lower by approx. 0.1. Concerning the interpretation of the obtained *β* and *x* values, the relatively low *β* seems to be fairly standard for low-molecular organic glasses, indicating that a large variety of structural movements (associated with a wide distribution of relaxation times) occurs in GSF. This is further supported by a rather low *x* value, which indicates a high dependence of the relaxation movements on the given structural state of the GSF glass, i.e., indicating a possible high cooperativity between the relaxing domains/entities. The latter is interesting, considering the lack of involvement of the polar groups in the intermolecular bonding [20], which drives the high surface diffusion. Hence, surface diffusion is apparently driven by significantly different mechanisms than structural relaxation—more research in this regard is definitely needed.

### 3.2. Kinetics of Crystallization in Amorphous GSF

The second phenomenon encountered during the GSF heating is cold crystallization (“cold” denotes crystal growth in a glassy state as opposed to liquid growth). Considering the complex crystallization behavior of GSF, each fully isolated crystallization peak was described individually to preserve the consistency with the previous partition to the crystallization pre-peak and main peak. The description of the crystallization pre-peak was performed only for low q^+^, where its signal could be reliably separated from the thermo-kinetic background. For all crystallization peaks, the tangential area-proportional baseline [59] was used to isolate the pure material response to the applied temperature program.

The kinetics of solid-state reactions and transformations (where crystallization falls in) is customarily described in terms of the following equations [60]:

(7) $$\Phi =\Delta H\cdot A\cdot e^{-E/RT}\cdot f\left(\alpha \right)$$

(8) $$f{\left(\alpha \right)}_{JMA}=m\left(1-\alpha \right){\left[-\ln\left(1-\alpha \right)\right]}^{1-\left(1/m\right)}$$

(9) $$f{\left(\alpha \right)}_{AC}=\alpha^{M}{\left(1-\alpha \right)}^{N}$$

where *Φ* is the raw measured heat flow, Δ*H* is the crystallization enthalpy, *A* is the pre-exponential factor, *E* is the activation energy of the macroscopically recorded crystal growth, *R* is the universal gas constant, *T* is temperature, *α* is conversion, and *f*(*α*) is a kinetic model function. The listed solid-state kinetic models (Equations (8) and (9)) correspond to the two most common and relevant crystallization models: the nucleation-growth Johnson–Mehl–Avrami [61,62,63,64] (JMA) model (Equation (6)) and the empirical autocatalytic Šesták–Berggren [59] (AC) model; *f*(*α*) kinetic exponents m, M, and N then stand for the parameters responsible for the asymmetry of the crystallization peak. The process of enumeration of Equations (7)–(9) customarily starts with the determination of Δ*H*; these values are listed in the Supplementary Materials. The second quantity to determine is the activation energy *E*. In this regard, two types of methods can be used—either the methods utilizing a single characteristic temperature (usually temperature corresponding to the maximum of the crystallization peak *T_p_*; hence, the *T_p_*-based methods) or the isoconversional methods that determine *E* in dependence on α (the latter are further classified as differential or integral) [65]. For the present data, the following methods will be utilized, representing all three types of evaluation: the *T_p_*-based Kissinger method [66] (Equation (10)), the differential isoconversional Friedman method [67] (Equation (11)), and the integral isoconversional Starink method [68] (Equation (12)).

(10) $$\ln\left(\frac{q^{+}}{T_{p}^{2}}\right)=-\frac{E}{RT_{p}}+const.$$

(11) $$\ln\left({\left[d\alpha /dt\right]}_{\alpha }\right)=-\frac{E_{\alpha }}{RT_{\alpha }}+const.$$

(12) $$\ln\left(\frac{q^{+}}{T_{p}^{1.92}}\right)=-1.008\frac{E_{\alpha }}{RT_{\alpha }}+const.$$

Note that the (d*α*/d*t*)*_α_*, *T_α_*, and *E_α_* are the conversion rate, temperature, and activation energy corresponding to the arbitrarily chosen values of conversion *α* (0.05–0.95 with a step of 0.05 for the present data). 

**Figure 5:**
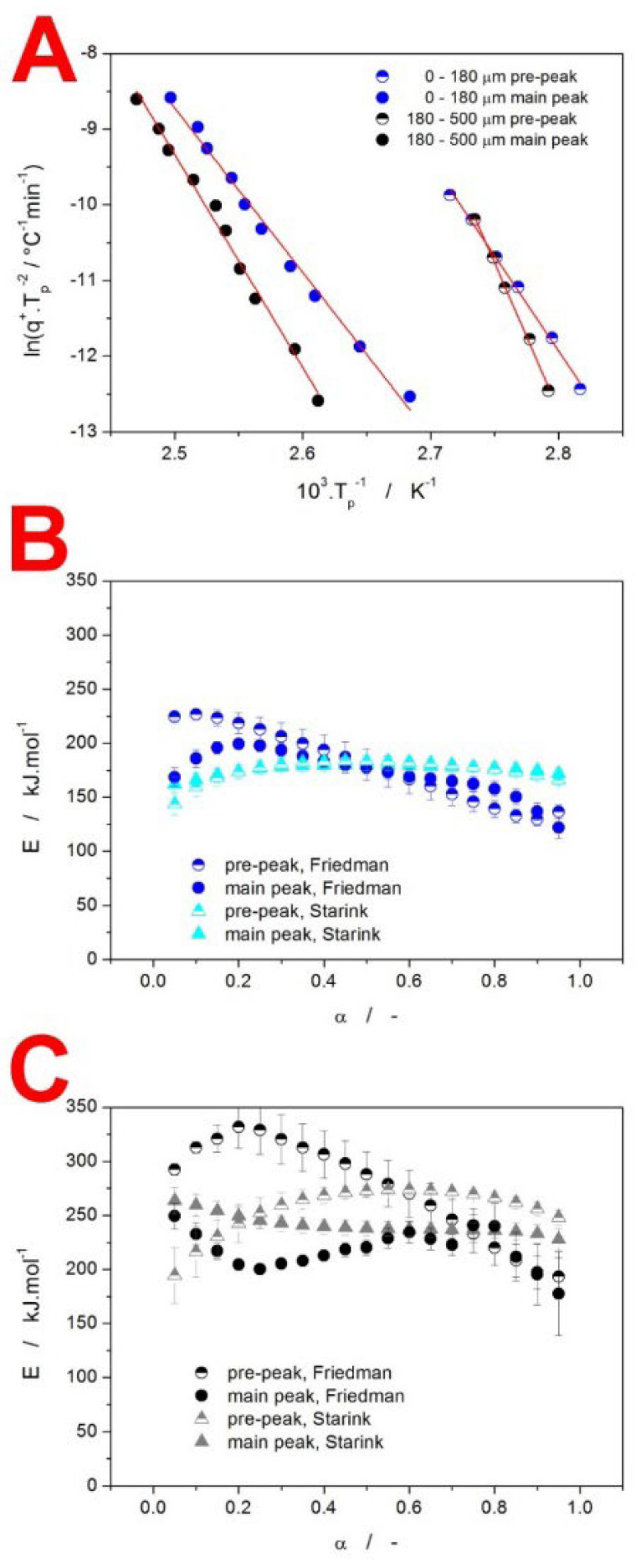
(**A**) Kissinger plot for the crystallization pre-peaks and main peaks—evaluation of the data from Figure 1. (**B**,**C**) *E*-*α* dependences determined by the Friedman and Starink methods for the crystallization pre-peaks and main peaks of 0–180 µm (**B**) and 180–500 µm (**C**) powders.

The evaluations of *E* by the above-mentioned methodologies are for the two GSF powders depicted in Figure 5. Starting with the Kissinger plot (Figure 5A), all dependencies are fairly linear, apart from the data obtained for the main crystallization peak of the 0–180 µm powder. As the linearity usually indicates a uniform and consistent crystal growth mechanism, the curvature observed for the main crystallization peak of the 0–180 µm powder can be most likely associated with the complexity of the crystal growth (as also indicated by the double-peak shape of the crystallization signal at low *q^+^*—see Figure 1A). The presence of the large amount of the crystalline phase formed within the crystallization pre-peak together with the increased time for the surface nucleation (provided by the low *q^+^*) accelerates the main crystal growth process and shifts its onset/peak to lower *T*. The gradually increased effect of these aspects with decreasing *q^+^* results in the curvature of the Kissinger dependence. Assuming the validity of linear fits for all Kissinger dependences from Figure 5A, the following values of activation energy and the pre-exponential factor are evaluated: *E* = 209.3 ± 6.4 kJ·mol^−1^ and log(*A*/s^−1^) = 28.00 (pre-peak, 0–180 µm); *E* = 180.4 ± 5.5 kJ·mol^−1^ and log(*A*/s^−1^) = 22.42 (main peak, 0–180 µm); *E* = 323.8 ± 9.3 kJ·mol^−1^ and log(*A*/s^−1^) = 44.53 (pre-peak, 180–500 µm); *E* = 233.8 ± 7.7 kJ·mol^−1^ and log(*A*/s^−1^) = 29.20 (pre-peak, 0–180 µm). Accounting for the curvature of the dependence of the 0–180 µm main peak, the following ranges of activation energies and pre-exponential factors characterize the q^+^-dependent *E*/*A* evolution: *E* ≈ 141–206 kJ·mol^−1^ and log(*A*/s^−1^) ≈ 16.9–25.9. In comparison with IMC [39] and NIM [38], the GSF activation energies for crystallization are significantly higher, especially for the growth in the coarse powder.

The isoconversional evaluations of E-α dependencies are shown in Figure 5B,C. The differential and integral isoconversional methods generally confirm the results of the Kissinger method. However, the Friedman method is here shown to be very sensitive to data distortions, as indicated by the increased variability of the *E*-*α* dependence. Overall, the present data show that the interpretation of the *E*-*α* dependences provided by the isoconversional methods can be tricky and often confusing for the unideal data, where different data-distortive effects can imitate true α-dependent variation of *E*. Hence, it is also the universal recommendation to take into account only the *E* values from the 0.3–0.7 α interval [65]. 

In the continuation of the Equation (1) enumeration, an appropriate kinetic model has to be chosen. As the JMA model would be preferred for the crystallization processes due to its physico-chemical meaningfulness, its applicability is usually checked first. In this regard, a quantified approach [69] utilizing the so-called master plot function z(α) can be recommended:

(13) $$z\left(\alpha \right)=f\left(\alpha \right)\cdot g\left(\alpha \right)=\Phi \cdot T^{2}$$

where g(α) is an integral form of the f(α) model. This simple transformation of the measured DSC signal *Φ* is evaluated with respect to the degree of conversion associated with the maximum of the z(α) function, i.e., *α_max,z_*. As has been recently pointed out [69], the following *α_max,z_* limits can be associated with the JMA model applicability: *α_max,z_* = 0.632 for 100% correlation, *α_max,z_* = 0.620–0.665 for r^2^ = 0.999 correlation, and *α_max,z_* = 0.585–0.705 for r^2^ = 0.995 correlation. Note that this pass-fail test is only the preliminary step for the JMA model applicability verification; a direct fit of the experimental data by the JMA model has to always follow. For the present GSF crystallization peaks, the following results (averaged over all heating scans performed for the given powder fraction) were obtained: *α_max,z_* = 0.561 ± 0.094 (pre-peak, 0–180 µm); *α_max,z_* = 0.678 ± 0.040 (main peak, 0–180 µm); *α_max,z_* = 0.530 ± 0.112 (pre-peak, 180–500 µm); *α_max,z_* = 0.659 ± 0.066 (main peak, 180–500 µm). As is apparent, this preliminary verification already rules out the JMA model for the pre-peaks of both powder fractions and indicates that a majority of the main crystallization peaks would (at best) fall into the r^2^ = 0.995 category. In addition, quite large *α_max,z_* errors indicate that the crystallization mechanism significantly changes with *q^+^*, which is inconsistent with the mathematical rigidity of the JMA model. For these reasons, the AC model will be used to describe the DSC data.

The direct curve-fitting of the DSC data by the combination of Equations (7) and (9) was done by means of the single-curve multivariate kinetic analysis (sc-MKA) [70] utilizing the *E* values obtained from the Kissinger plot:

(14) $$RSS=\sum_{j=1}^{n}\sum_{k=First_{j}i}^{Last_{j}}w_{j,k}{\left(Y\exp_{j,k}-Ycal_{j,k}\right)}^{2}$$

(15) $$w_{j}=\frac{1}{{\left|{\left[d\alpha /dt\right]}_{\max}\right|}_{j}+{\left|{\left[d\alpha /dt\right]}_{\min}\right|}_{j}}$$

where *RSS* is the sum of squared residue, *n* is number of measurements, *j* is index of the given measurement, *First_j_* is the index of the first point of the given curve, *Last_j_* is the index of the last point of the given curve, *Yexp_j,k_* is the experimental value of the point *k* of curve *j*, *Ycal_j,k_* is the calculated value of the point *k* of curve *j*, and *w_j_* is the weighting factor for curve *j*. However, the first round of non-linear optimizations has shown that a single-process transformation mechanism is not sufficient to adequately describe the experimental data—this finding was found to be valid for practically all data peaks, even those manifesting as an apparent single peak with detectable shoulders. For this reason, the double process kinetics with both sub-peaks being expressed by the AC model was adopted:

(16) $$\Phi =\Delta H_{1}\cdot A_{1}\cdot e^{-E_{1}/RT}\cdot \alpha_{1}^{M_{1}}{\left(1-\alpha_{1}\right)}^{N_{1}}+\Delta H_{2}\cdot A_{2}\cdot e^{-E_{2}/RT}\cdot \alpha_{2}^{M_{2}}{\left(1-\alpha_{2}\right)}^{N_{2}}$$

(17) $$\alpha =\frac{\Delta H_{1}}{\Delta H}\alpha_{1}+\frac{\Delta H_{2}}{\Delta H}\alpha_{2}$$

where the indices 1 and 2 denote the given sub-processes. The results are listed in the Supplementary Materials. Overall, the very high correlation coefficients in the case of the main crystallization peaks indicate sufficient precision and accuracy for the data to be used in the kinetic modeling and predictions [43,70]. The significantly worse correlation coefficients obtained in the case of the crystallization pre-peaks (especially those obtained at high *q^+^*) can be attributed to the overlap with the glass transition effect, where the subtraction of the thermo-kinetic background was not precise enough. For these data, the simplified modeling/prediction approach based only on the *E* and *A* values would be recommended [70]. The most important conclusion resulting from the tabulated sc-MKA data is that the crystal growth mechanism is significantly temperature-dependent, and for the kinetic predictions extrapolated to the lower temperatures, the mathematic description determined for the *q^+^* = 0.5 °C·min^−1^ would be the most relevant and accurate to use [43,70].

### 3.3. Kinetics of GSF Thermal Decomposition

**Figure 6:**
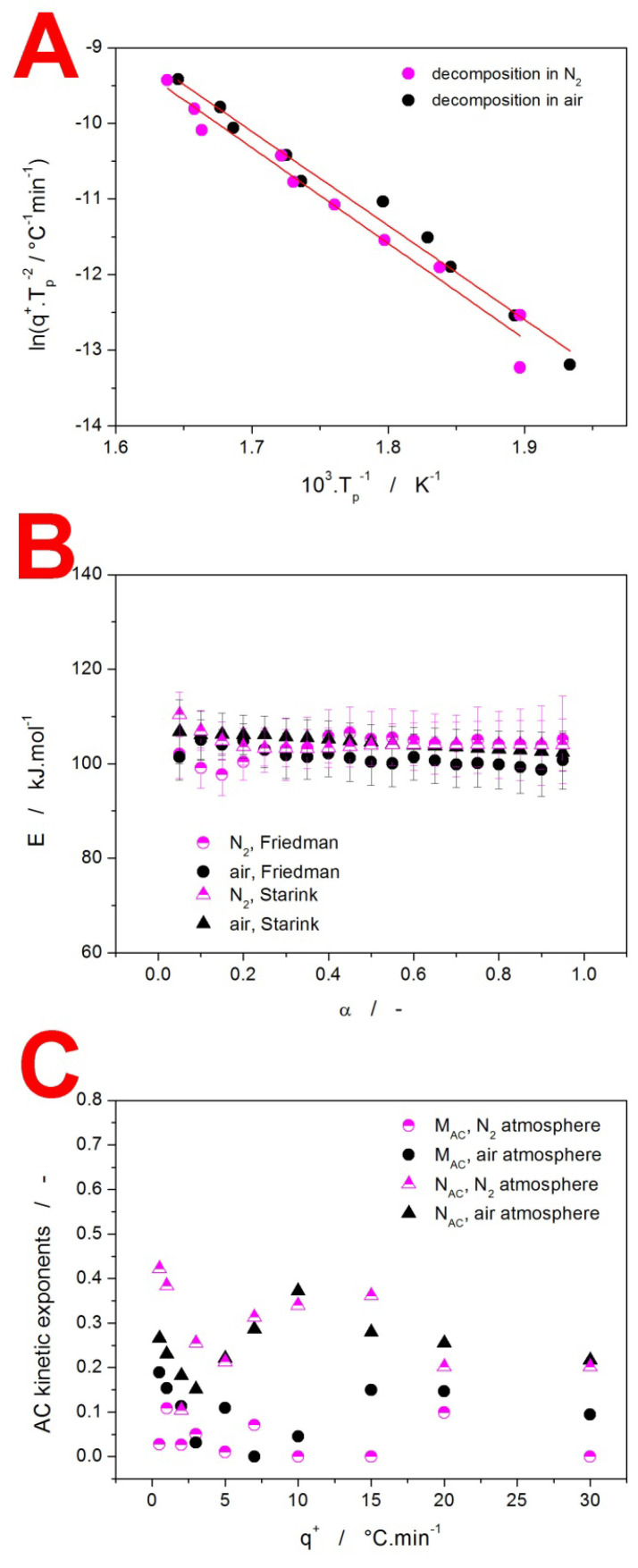
(**A**) Kissinger plot for the decomposition in N_2_ and in air. (**B**) *E*-*α* dependences determined by the Friedman and Starink methods for the decomposition data. (**C**) AC kinetic exponents evaluated by the sc-MKA method for the decomposition data.

The thermogravimetric curves of high-*T* GSF decomposition (Figure 3A,B) were treated in a methodological manner similar to the previously discussed crystallization data. The Kissinger dependences (Equation (10)) corresponding to the decomposition in two reaction atmospheres (N_2_ and air) are depicted in Figure 6A. The mutual position of the two dependences indicates that the decomposition in air may proceed very slightly faster but with practically similar activation energy: *E_decomp_* = 104.0 ± 4.2 kJ·mol^−1^ and log(*A*/s^−1^) = 7.48 (air); *E_decomp_* = 105.8 ± 5.8 kJ·mol^−1^ and log(*A*/s^−1^) = 7.53 (N_2_). Very similar activation energy values were also obtained by the isoconversional methods (Equations (11) and (12))—see Figure 6B. Contrary to the crystallization data, the thermal decomposition shows remarkably constant E-α dependences, indicating the uniformity and reproducibility of the decomposition reaction mechanism. As the physical meaningfulness of the nucleation-growth kinetics is questionable for the thermal decomposition, the AC model was used as a starting point for the sc-MKA-based description of the TGA data shown in Figure 3A,B. The mass loss associated with the decomposition was indeed described by a single-step autocatalytic reaction mechanism with exceptionally high accuracy (average r^2^ > 0.9999). The model-based kinetics is for the present TGA data expressed via the evolution of the *M* and *N* AC kinetic exponents—see Figure 6C. 

Despite the empirical nature of the AC model, its exponents can be interpreted as follows. The very low M values indicate a low degree of autocatalytic acceleration of the GSF decomposition, which means that the decomposition behavior can be interpreted in terms of the *n*th-order reaction model. However, as the decomposition usually consists of a series of reaction steps that are not known in the case of GSF, a rather mechanistic interpretation should be adopted. Namely, the lower the reaction order (expressed by *N* in the AC model), the slower onset, and the steeper endset characterize the reaction [59,60]. Furthermore, the data from Figure 6C show that there is very little difference even between the model-based kinetics for the decompositions proceeding under the two tested atmospheres—the presence of oxygen has thus no practical effect, and the sample burning (formation of char or tar) can be ruled out. Kinetic predictions-wise, clear trends occur for the majority of the kinetic exponents at the lowest *q^+^* (0.5–3 °C·min^−1^), which is paramount for the eventual extrapolation of the *M* and *N* values during the modeling of the long-term (low-*T*) decomposition [70]. Compared to the decomposition of IMC [39] and NIM [38], GSF exhibits very similar activation energy for the decomposition process (within 5% of both other drugs). On the other hand, the decomposition of GSF exhibits the slowest reaction/decomposition onset (lowest *N* as well as *M*). Temperature-wise, GSF decomposes only 10–20 °C above IMC and NIM (despite having a significantly higher melting point). The coincidence of the GSF melting temperature and the onset of decomposition measured at very low *q^+^* indicates that evaporation may play a significant role in the TGA-detected mass loss.

### 3.4. Mutual Relationships between Structural Relaxation, Microscopic and Macroscopic Crystal Growth

**Figure 7:**
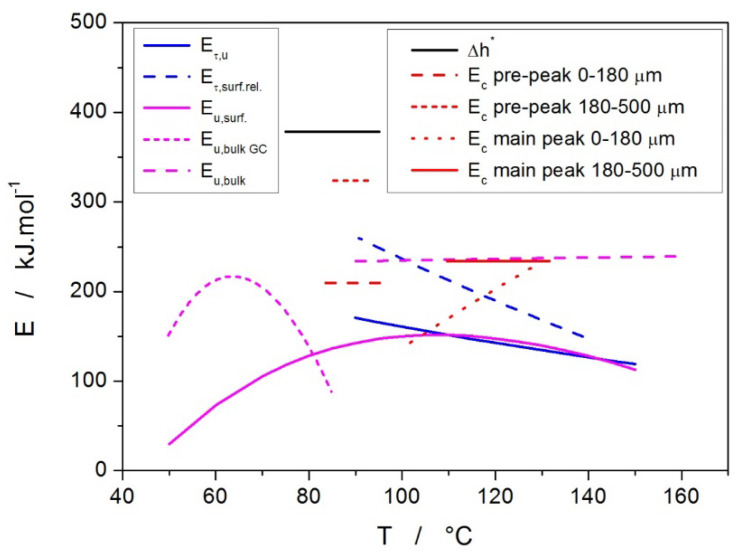
Activation energies of DSC-measured structural relaxation and crystallization (this work), microscopically monitored crystal growth modes [71], and characteristic relaxation/growth times τ [72].

In the previous studies on IMC [39] and NIM [38], it was found to be very useful to assess the mutual relationships between the structural relaxation and crystal growth phenomena on the basis of the activation energies corresponding to the given processes. Since the present study reports only on the macroscopic manifestation of the thermally induced phenomena, the data for the microscopically monitored crystal growth rates were taken from the literature [71,72]. Where possible, the temperature-dependent activation energies were determined using the following expressions:

(18) $$\frac{d\ln\left(u_{G}\right)}{d\left(1/T\right)}=-\frac{E_{u}}{R}$$

(19) $$\frac{d\ln\left(\tau \right)}{d\left(1/T\right)}=-\frac{E_{\tau }}{R}$$

where *u_G_* is the microscopically determined crystal growth rate (surface, bulk, or diffusionless GC growth), and *τ* represents either the characteristic surface crystallization time *τ_u_* or the surface relaxation time *τ_surf.rel._*. The actual temperature dependencies were determined by fitting the corresponding data (*u_G_*-*T* or *τ*-*T* dependencies) by second- or third-order polynomial functions and calculating the derivations (in accordance with Equations (18) and (19)) of these fits. The complete set of *T*-dependent activation energies is shown in Figure 7; the only missing data curve is that for the thermal decomposition, which would stand at ~105 kJ·mol^−1^ in the 200–350 °C temperature range. 

Starting with the structural relaxation-related data, the most important datum is probably the relation between ∆h* and *E_c_* obtained for the two crystallization pre-peaks. As the pre-peak crystallization appears to be initiated by the glass transition, one might further expect the glass transition to be the rate-determining step, i.e., the activation energies to be similar for the two processes. This is, however, not the case—the 180–500 µm pre-peak has lower *E_c_* by approx. 60 kJ·mol^−1^, and the *E_c_* of the 0–180 µm pre-peak is lower by approx. 170 kJ·mol^−1^. Hence, the crystal growth (and possibly nucleation as well) is significantly slower at these temperatures, and the glass transition effect is indeed responsible only for the initiation of the crystalline phase formation (which in turn means that the growth is not determined by the surface self-diffusion). Noteworthy is, however, the correspondence of *E_c_* attributed to the 0–180 µm pre-peak and maximum *E_u,bulk GC_*, which might indicate an influence of the GC growth on the formation of the defects-located nuclei that start to grow later, with the glass transition initiation. Another relationship can be sought between ∆h* and the *E_τ,surf.rel._* quantity, which corresponds to the surface relaxation time. As was suggested in [73], the free energy barrier for the relaxation motions in bulk should be double that near a free surface. Based on this assumption (consistent with the “random first-order transition model”), the *τ_surf.rel._* was predicted in [72] for GSF; by applying Equation (19) to those data, we have determined the *E_τ,surf.rel._* activation energy (see Figure 7), which is indeed roughly half of ∆h* (activation energy for bulk structural relaxation).

Moving to the surface crystal growth, the derivation of the microscopically determined *u_G_*-*T* dependence [71] (according to Equation (18)) gave an activation energy *E_u,surf_* that is very consistent with the activation energy determined for the surface crystallization from the *τ_u_*-*T* dependence reported in [72]. Interestingly, the activation energy for the main peak of the macroscopic (DSC-monitored) crystallization in the 0–180 µm powder transitions from *E_u,surf_* to *E_u,bulk_* as *T* increases. The inclination of the crystallization mechanism to that akin to bulk crystallization (despite the high number of surface defects in the finely ground powder) may be caused, e.g., by self-healing at higher *T*, by the negligible amount of crystalline phase formed during the crystallization pre-peak at high (*q^+^*), or by the energy surplus (again achieved during fast heating) that may favor the (possibly more numerous) bulk-located growth centers. For the coarse GSF powder (180–500 µm), the macroscopic crystallization activation energy obtained for the main process is in the whole temperature range, practically identical to the microscopically evaluated *E_u,bulk_*, which is in agreement with the low number of mechanical defects in the 180–500 µm powder that could act as surface crystallization centers. These findings indicate (similarly to [38,39]) that it is, in fact, the nucleation (and not the growth itself) that is key for the understanding of not only polymorphic preference but also of the favored formation of the crystalline phase from different types of crystal growth centers (surface/volume defects and micro-cracks, surface and volume nuclei formed during the glass quench or during the consequent heating of the glass). 

## 4. Experimental

The bulk glassy GSF was prepared from the as-purchased crystalline product (Sigma-Aldrich, Prague, Czech Republic, purity > 97%) by a standard melt-quench method: The crystalline powder was placed in a glass vial, liquefied in an oil bath at a temperature slightly above its melting point (~215 °C), and quenched (still in the vial) in cold water (~10 °C). The amorphous ingot of yellowish color was then taken out of the vial and lightly ground using an agate mortar and pestle. Two batches of powder with defined particle sizes were prepared from the ground bulk glass via sieving through the sieves (Retsch, Prague, Czech Republic) with known mesh: 0–180 µm and 180–500 µm (i.e., the 180 µm and 500 µm sieves were used). The powders were stored in a cooled (10 °C) dark desiccator; the absolute majority of the thermo-analytical measurements were performed within 96 h of the amorphous phase preparation. Note that no traces of crystalline phase were detected by means of Raman spectroscopy even after 14 days of storage. Similarly, no change in the crystallization behavior (that could indicate nucleation during storage) was found during the repeated calorimetric monitoring after 14 days.

The thermally induced phase transitions in the amorphous GSF were studied by means of differential scanning calorimetry (DSC), using the heat-flow Q2000 DSC instrument (TA Instruments, New Castle, DE, USA) equipped with an auto-sampler, an RCS90 cooling accessory, and T-zero technology. Calibration of the DSC was done using the In, Zn, and H_2_O standards. All DSC measurements were performed for the two powder size fractions; the samples with masses between 2.5 and 3.5 mg were accurately (to 0.01 mg) weighted into low-mass Al pans and hermetically sealed (the measurements were thus performed in a static air atmosphere). For the crystallization/characterization measurements, simple heating scans were performed (always for a fresh sample) in the 10–250 °C temperature range at heating rates *q^+^* = 0.5, 1, 2, 3, 5, 7, 10 15, 20, and 30 °C·min^−1^. The structural relaxation measurements were performed as two sets of specific cyclic experiments: the constant ratio (CR) cycles [55], and the constant heating rate (CHR) cycles [74], where the sample was repeatedly cooled and heated through the glass transition region. During the CR cycles, the following cooling rates were used for the cooling steps: *q^−^* = 0.5, 1, 2, 3, 5, 7, 10, 15, 20, and 30 °C·min^−1^; the consequent heating steps were performed at *q^+^* similar to the *q^−^* of the preceding cooling step. During the CHR cycles, again, identical *q^−^* rates were used as in the CR cycles, but the heating rate was constant in all CHR cycles *q^+^* = 10 °C·min^−1^. The temperature range for both types of cycles was 10–97 °C; for each powder size fraction, the identical sample was used for all CR and CHR cycles to eliminate instrumental artifacts and increase the precision of the measurements. The reproducibility of the crystallization and relaxation experiments was confirmed for several randomly selected measurements; also, the reproducibility checks repeated after 14 days of material storage still showed perfect overlap with the previously measured data.

The high-temperature degradation/decomposition of GSF was monitored by means of thermogravimetry using the STA (TGA) 449 F5 Jupiter instrument (Netzsch, Selb, Germany) equipped with a DSC/TG holder. The STA measurements were performed as a series of heating scans at *q^+^* = 0.5, 1, 2, 3, 5, 7, 10, 15, 20, and 30 °C·min^−1^ with each heating scan performed for a fresh sample. Since the GSF decomposition proceeds well above the melting point *T_m_* ≈ 215 °C, the initial form of the GSF powder does not play any role. Hence, only the 180–500 µm powder was used. On the other hand, the atmosphere may be relevant for the decompositions, so two series of STA measurements were performed—one in pure N_2_ and one in air (gas flow rate 50 mL·min^−1^ in both cases). The sample masses varied between 1.5 and 2.5 mg; open Al pans were used as the sample vessels. 

Additional characterization of the amorphous and crystalline GSF samples was done by means of Raman microscope DXR2 (Nicolet, Thermo Fisher Scientific, Prague, Czech Republic) equipped with the 785 nm excitation diode laser (30 mW, laser spot size 1.6 μm) and CCD detector. The Raman spectra were collected under the following conditions: 25 mW laser power on the sample, 1s duration of a single scan, and 50 scans summed in one spectrum. The X-ray diffraction (XRD) pattern of the crystallized sample was obtained by the XRD instrument (Empyrean Malvern Panalytical, Malvern, UK) in the 5–80° range, positioned on a holder. The optical microscope iScope PLMi (Euromex, Arnhem, Netherlands), equipped with high-quality ×20 and ×40 objectives and a Moticam visual camera, was used in transmission mode to observe the morphology of the formed crystallites.

## 5. Conclusions

Thermo-analytical (DSC, TGA), microscopic, spectroscopic (Raman microscopy), and diffraction (XRD) methods were used to explore the processes of structural relaxation, crystal growth, and thermal decomposition in amorphous GSF. The kinetics of the structural relaxation movements was modeled using the following set of TNM relaxation parameters: ∆h* = 395 ± 25 kJ·mol^−1^, ln(*A*/s) = −131 ± 5.5, *x* = 0.28 ± 0.01, *β* = 0.405 ± 0.005. In general, the amorphization of GSF is not a problem (simple quench into cold water was sufficient to glassify approx. 1 g of molten material in a vial), and the amorphous material is very stable even at temperatures near its *T_g_* ≈ 80 °C. However, GSF is very susceptible to the formation of crystalline phase from mechanical defects—sharp edges, micro-cracks, etc. Powdered amorphous GSF rapidly crystallizes in the glass transition region; the amount of formed crystalline phase increases with decreasing *q^+^* and decreasing particle size. The glass transition is, however, only initiating the crystal growth, which itself is the slower process of the two. In addition, the sub-*T_g_* growth mode (recognized in GSF [10,20,71]) may be associated with the formation of defects-located nuclei, from which the crystal growth in the glass transition region initiates. As was proven during the sample preparation for the structural relaxation measurements, rapid initial heating above *T_g_* self-heals/seals the nucleation centers, which remarkably stabilizes the powdered amorphous GSF (this procedure may be crucial for successful processing and long-term storage of amorphous GSF). The main crystallization process occurs for the amorphous GSF in the 115–135 °C range; the identical polymorphic phase (dominant form I) is a product of both crystallization processes (pre-peak in the *T_g_* range and the main peak near 120 °C). The crystallization of the fine powder proceeds with temperature-dependent activation energy, where at low *T*, *E_c_* ≈ *E_u,surf_*, and at high *T*, *E_c_* ≈ *E_u,bulk_*. For the coarse GSF powder, the activation energy is constant in the whole temperature range: *E_c_* ≈ *E_u,bulk_* ≈ 235 kJ·mol^−1^. The absolute majority of the crystallization data does not follow the simple nucleation-growth kinetics; a complex overlap of two autocatalytic kinetics was used to accurately describe the DSC crystallization data. Thermal decomposition of GSF proceeds identically in N_2_ and in air atmospheres; the activation energy of the process is *E_decomp_* ≈ 106 kJ·mol^−1^. The coincidence of the GSF melting temperature and the onset of decomposition measured at very low *q^+^* indicates that evaporation may play a significant role in the TGA-detected mass loss.

## Data Availability

The original data are available on request from the corresponding author.

## Supplementary Materials

The following supporting information can be downloaded at: https://www.mdpi.com/article/10.3390/molecules29071516/s1, Figures S1–S8: zoomed-in and zoomed-out versions of graphs in Figure 1. Table S1: Characteristic temperatures and enthalpies evaluated from the DSC curves.; Table S2: Parameters of the standard kinetic equation for crystallization data.

## References

[1] Grandner J., Cacho R., Tang Y., Houk K. (2016). Mechanism of the p450-catalyzed oxidative cyclization in the biosynthesis of griseofulvin. ACS Catal..

[2] Duldulao P., Ortega A., Delgadillo X. (2019). Mycotic and bacterial infections. Clin. Colon Rectal Surg..

[3] Otto D., Otto A., Villiers M. (2022). In vitro skin delivery of griseofulvin by layer-by-layer nanocoated emulsions stabilized by whey protein and polysaccharides. Pharmaceutics.

[4] Schmeel L., Schmeel F., Kim Y., Blaum-Feder S., Schmidt-Wolf I. (2017). Griseofulvin efficiently induces apoptosis in in vitro treatment of lymphoma and multiple myeloma. Anticancer Res..

[5] Grigalunas M., Patil S., Krzyzanowski A., Pahl A., Flegel J., Schölermannet B., Xie J., Sievers S., Ziegler S., Waldmann H. (2022). Unprecedented combination of polyketide natural product fragments identifies the new hedgehog signaling pathway inhibitor grismonone. Chem. Eur. J..

[6] Smith C., Jerkovic A., Truong T., Foote S., McCarthy J., McMorran B. (2017). Griseofulvin impairs intraerythrocytic growth of plasmodium falciparum through ferrochelatase inhibition but lacks activity in an experimental human infection study. Sci. Rep..

[7] Ricci A., Pierro E., Marcacci M., Ventura P. (2021). Mechanisms of neuronal damage in acute hepatic porphyrias. Diagnostics.

[8] Hee-Jeong K., Sang-Do Y. (2015). Liquid antisolvent crystallization of griseofulvin from organic solutions. Chem. Eng. Res. Des..

[9] Chen H., Wang C., Sun C.C. (2019). Profoundly Improved Plasticity and Tabletability of Griseofulvin by in Situ Solvation and Desolvation during Spherical Crystallization. Cryst. Growth Des..

[10] Shi Q., Zhang C., Su Y., Zhang J., Zhou D., Cai T. (2017). Acceleration of Crystal Growth of Amorphous Griseofulvin by Low-Concentration Poly(ethylene oxide): Aspects of Crystallization Kinetics and Molecular Mobility. Mol. Pharm..

[11] Rahman M., Ahmad S., Tarabokija J., Parker N., Bilgili E. (2020). Spray-Dried Amorphous Solid Dispersions of Griseofulvin in HPC/Soluplus/SDS: Elucidating the Multifaceted Impact of SDS as a Minor Component. Pharmaceutics.

[12] Chakravarty P., Famili A., Nagapudi K., Al-Sayah M. (2019). Using supercritical fluid technology as a green alternative during the preparation of drug delivery systems. Pharmaceutics.

[13] Kumar R., Dalvi S., Siril P. (2020). Nanoparticle-based drugs and formulations: Current status and emerging applications. ACS Appl. Nano Mater..

[14] Liu Q., Mao L., Liu C., Yin J., Zhu X., Chen D. (2021). Continuous synthesis of polymer-coated drug nanoparticles by heterogeneous nucleation in a hollow-fiber membrane module. Ind. Eng. Chem. Res..

[15] Bhujbal S., Mitra B., Jain U., Gong Y., Agrawal A., Karkiet S., Taylor L.S., Kumar S., Zhou Q.T. (2021). Pharmaceutical amorphous solid dispersion: A review of manufacturing strategies. Acta Pharm. Sin. B.

[16] Willart J.F., Dudognon E., Mahieu A., Eddleston M., Jones W., Descamps M. (2017). The role of cracks in the crystal nucleation process of amorphous griseofulvin. Eur. Phys. J. Spec. Top..

[17] Willart J.F., Carpentier L., Danède F., Descamps M. (2012). Solid-state vitrification of crystalline griseofulvin by mechanical milling. J. Pharm. Sci..

[18] Brian C.W., Yu L. (2013). Surface self-diffusion of organic glasses. J. Phys. Chem. A.

[19] Zhu L., Jona J., Nagapudi K., Wu T. (2010). Fast surface crystallization of amorphous griseofulvin below Tg. Pharm. Res..

[20] Huang C., Ruan S., Cai T., Yu L. (2017). Fast Surface Diffusion and Crystallization of Amorphous Griseofulvin. J. Phys. Chem. B.

[21] Zhou D., Zhang G.G.Z., Law D., Grant D.J.W., Schmitt E.A. (2008). Thermodynamics, Molecular Mobility and Crystallization Kinetics of Amorphous Griseofulvin. Mol. Pharm..

[22] Trenfield S., Awad A., Goyanes Á., Gaisford S., Basit A. (2018). 3d printing pharmaceuticals: Drug development to frontline care. Trends Pharmacol. Sci..

[23] Vithani K., Goyanes Á., Jannin V., Basit A., Gaisford S., Boyd B. (2019). A proof of concept for 3d printing of solid lipid-based formulations of poorly water-soluble drugs to control formulation dispersion kinetics. Pharm. Res..

[24] Hong X., Han X., Li X., Li J., Wang Z., Zheng A. (2021). Binder jet 3d printing of compound lev-pn dispersible tablets: An innovative approach for fabricating drug systems with multicompartmental structures. Pharmaceutics.

[25] Mathew E., Pitzanti G., Larrañeta E., Lamprou D. (2020). 3d printing of pharmaceuticals and drug delivery devices. Pharmaceutics.

[26] Buyukgoz G., Kossor C., Davé R. (2021). Enhanced supersaturation via fusion-assisted amorphization during fdm 3d printing of crystalline poorly soluble drug loaded filaments. Pharmaceutics.

[27] Renterghem J., Vervaet C., Beer T. (2017). Rheological characterization of molten polymer-drug dispersions as a predictive tool for pharmaceutical hot-melt extrusion processability. Pharm. Res..

[28] Shu L., Tian Y., Jones D., Andrews G. (2016). Optimising drug solubilisation in amorphous polymer dispersions: Rational selection of hot-melt extrusion processing parameters. Aaps Pharmscitech.

[29] Figueroa F., Espinell J., Hernández M., López-Mejías V., Stelzer T. (2022). Polymorphic phase transformations in crystalline solid dispersions: The combined effect of pressure and temperature. Cryst. Growth Des..

[30] Gupta S., Solanki N., Serajuddin A. (2015). Investigation of thermal and viscoelastic properties of polymers relevant to hot melt extrusion, iv: Affinisol™ hpmc hme polymers. Aaps Pharmscitech.

[31] Chamberlain R., Windolf H., Geissler S., Quodbach J., Breitkreutz J. (2022). Precise dosing of pramipexole for low-dosed filament production by hot melt extrusion applying various feeding methods. Pharmaceutics.

[32] Ma X., Müller F., Huang S., Lowinger M., Liu X., Schooleret R., Williams R.O. (2020). Influence of carbamazepine dihydrate on the preparation of amorphous solid dispersions by hot melt extrusion. Pharmaceutics.

[33] Yi S., Wang J., Liu Y., Ma R., Gao Q., Liuet S., Xiong S. (2019). Novel hot melt extruded matrices of hydroxypropyl cellulose and amorphous felodipine–plasticized hydroxypropyl methylcellulose as controlled release systems. Aaps Pharmscitech.

[34] Kawakami K. (2019). Crystallization Tendency of Pharmaceutical Glasses: Relevance to Compound Properties, Impact of Formulation Process, and Implications for Design of Amorphous Solid Dispersions. Pharmaceutics.

[35] Ediger M.D., Harrowell P., Yu L. (2008). Crystal growth kinetics exhibit a fragility-dependent decoupling from viscosity. J. Chem. Phys..

[36] Musumeci D., Hasebe M., Yu L. (2016). Crystallization of Organic Glasses: How Does Liquid Flow Damage Surface Crystal Growth? Cryst. Growth Des..

[37] Swallen S.F., Ediger M.D. (2011). Self-diffusion of the amorphous pharmaceutical indomethacin near Tg. Soft Matter.

[38] Svoboda R., Macháčková J., Nevyhoštěná M., Komersová A. (2024). Thermal stability of amorphous nimesulide: From glass formation to crystal growth and thermal degradation. Phys. Chem Chem. Phys..

[39] Svoboda R., Košťálová D., Krbal M., Komersová A. (2022). Indomethacin: The Interplay between Structural Relaxation, Viscous Flow and Crystal Growth. Molecules.

[40] Svoboda R., Málek J. (2015). Evaluation of glass-stability criteria for chalcogenide glasses: Effect of experimental conditions. J. Non-Cryst. Sol..

[41] Svoboda R., Kincl M., Málek J. (2015). Thermal characterization of Se-Te thin films. J. Alloys Compd..

[42] Ou X., Li S., Chen Y., Rong H., Li A., Lu M. (2022). Polymerphism in Griseofulvin: New Story between an Old Drug and Polyethylene Glycol. Cryst. Growth Des..

[43] Svoboda R., Koutná N., Košťálová D., Krbal M., Komersová A. (2023). Indomethacin: Effect of Diffusionless Crystal Growth on Thermal Stability during Long-Term Storage. Molecules.

[44] Martin J.D., Hillis B.G., Hou F. (2020). Transition Zone Theory Compared to Standard Models: Reexamining the Theory of Crystal Growth from Melts. J. Phys. Chem. C.

[45] Berthier L., Chandler D., Garrahan J.P. (2005). Length scale for the onset of Fickian diffusion in supercooled liquids. Europhys. Lett..

[46] Chong S.-H., Kob W. (2009). Coupling and Decoupling between Translational and Rotational Dynamics in a Supercooled Molecular Liquid. Phys. Rev. Lett..

[47] Scherer G.W. (1990). Theories of relaxation. J. Non-Cryst. Sol..

[48] Hodge I.M. (1994). Enthalpy relaxation and recovery in amorphous materials. J. Non-Cryst. Sol..

[49] Rozo J.I.J., Zarow A., Zhou B., Pinal R., Iqbal Z., Romañach R.J. (2011). Complementary near-infrared and raman chemical imaging of pharmaceutical thin films. J. Pharm. Sci..

[50] Smith G.P.S., Huff G.S., Gordon K.C. (2016). Investigating Crystallinity Using Low Frequency Raman Spectroscopy: Applications in Pharmaceutical Analysis. Spectroscopy.

[51] Tool A.Q. (1946). Relation between inelastic deformability and thermal expansion of glass in its annealing range. J. Am. Ceram. Soc..

[52] Narayanaswamy O.S. (1971). A Model of Structural Relaxation in Glass. J. Am. Ceram. Soc..

[53] Moynihan C.T., Easteal A.J., De Bolt M.A., Tucker J. (1976). Dependence of the Fictive Temperature of Glass on Cooling Rate. J. Am. Ceram. Soc..

[54] Svoboda R., Málek J. (2013). Description of enthalpy relaxation dynamics in terms of TNM model. J. Non-Cryst. Solids.

[55] Svoboda R. (2014). Utilization of “q+/q−=const.” DSC cycles for physical aging studies. Eur. Polym. J..

[56] Svoboda R. (2015). Novel equation to determine activation energy of enthalpy relaxation. J. Therm. Anal..

[57] Svoboda R., Málek J. (2012). Enthalpy relaxation in Ge–Se glassy system. J. Therm. Anal..

[58] Hodge I.M., Berens A.R. (1982). Effects of annealing and prior history on enthalpy relaxation in glassy polymers. 2. Mathematical modeling. Macromolecules.

[59] Šesták J. (1984). Thermophysical Properties of Solids, Their Measurements and Theoretical Analysis.

[60] Šesták J. (2005). Science of Heat and Thermophysical Studies: A Generalized Approach to Thermal Analysis.

[61] Johnson W.A., Mehl K.F. (1939). Reaction kinetics in processes of nucleation and growth. Trans. Am. Inst. Min. (Met.) Eng..

[62] Avrami M. (1939). Kinetics of phase change I–general theory. J. Chem. Phys..

[63] Avrami M. (1940). Kinetics of phase change. II–transformation-time relations for random distribution of nuclei. J. Chem. Phys..

[64] Avrami M. (1941). Granulation, phase change, and microstructure—Kinetics of phase change III. J. Chem. Phys..

[65] Vyazovkin S., Burnham A.K., Criado J.M., Pérez-Maqueda L.A., Popescu C., Sbirrazzuoli N. (2011). ICATC Kinetics Committee recommendations for performing kinetic computations on thermal analysis data. Thermochim. Acta.

[66] Kissinger H.E. (1957). Reaction kinetics in differential thermal analysis. Anal.Chem..

[67] Friedman H.L. (1964). Kinetics of Thermal Degradation of Char-Forming Plastics from Thermogravimetry. Application to a Phenolic Plastic.

[68] Starink M.J. (2003). The determination of activation energy from linear heating rate experiments: A comparison of the accuracy of isoconversion methods. Thermochim. Acta.

[69] Svoboda R. (2021). Crystallization of glasses—When to use the Johnson-Mehl-Avrami kinetics?. J. Eur. Ceram. Soc..

[70] Svoboda R., Chovanec J., Slang S., Beneš L., Konrád P. (2021). Single-curve multivariate kinetic analysis: Application to the crystallization of commercial Fe-Si-Cr-B amorphous alloys. J. Alloys Compd..

[71] Shi Q., Cai T. (2016). Fast Crystal Growth of Amorphous Griseofulvin: Relations between Bulk and Surface Growth Modes. Cryst. Growth Des..

[72] Li F., Xin J., Shi Q. (2021). Diffusion-controlled and “diffusionless” crystal growth: Relation between liquid dynamics and growth kinetics of griseofulvin. J. Appl. Cryst..

[73] Stevenson J.D., Wolynes P.G. (2008). On the surface of glasses. J. Chem. Phys..

[74] Svoboda R. (2015). Utilization of constant heating rate DSC cycles for enthalpy relaxation studies and their influenceability by erroneous experimental operations. J. Non-Cryst. Solids.

