# Supplementary material for: Thermo-Structural Characterization of Phase Transitions in Amorphous Griseofulvin: From Sub-Tg Relaxation and Crystal Growth to High-Temperature Decomposition
Source: Molecules. 2024 Mar 28;29(7):1516. doi: 10.3390/molecules29071516 (PMC11013327; doi:10.3390/molecules29071516)
Supplement: Supplementary file 1 [file molecules-29-01516-s001.zip › molecules-2916164-supplementary.pdf]

Part 1

Zoomed-in and zoomed-out graphs corresponding to those shown in Figure 1.

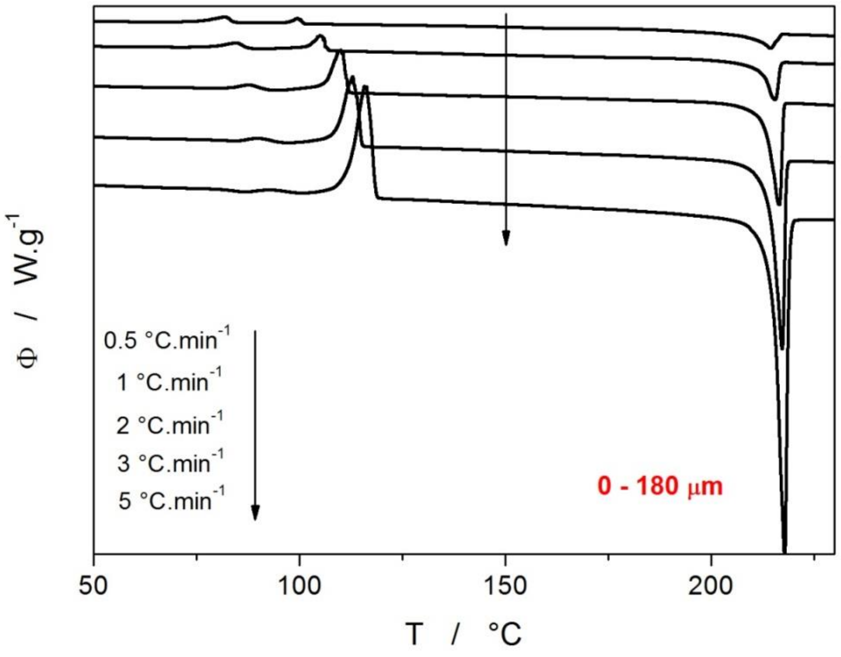

Figure S1: Zoomed-out data from Figure 1.

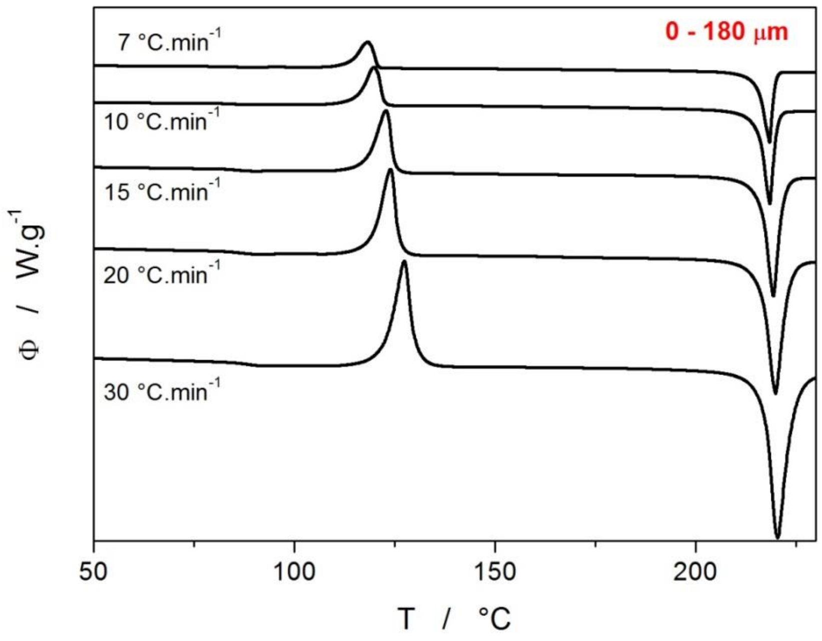

Figure S2: Zoomed-out data from Figure 1.

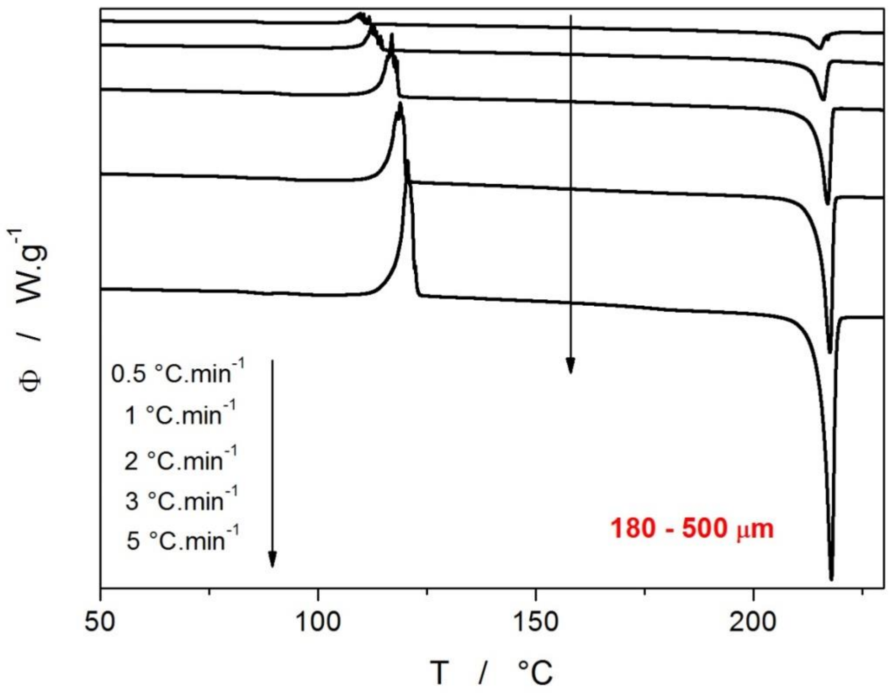

Figure S3: Zoomed-out data from Figure 1.

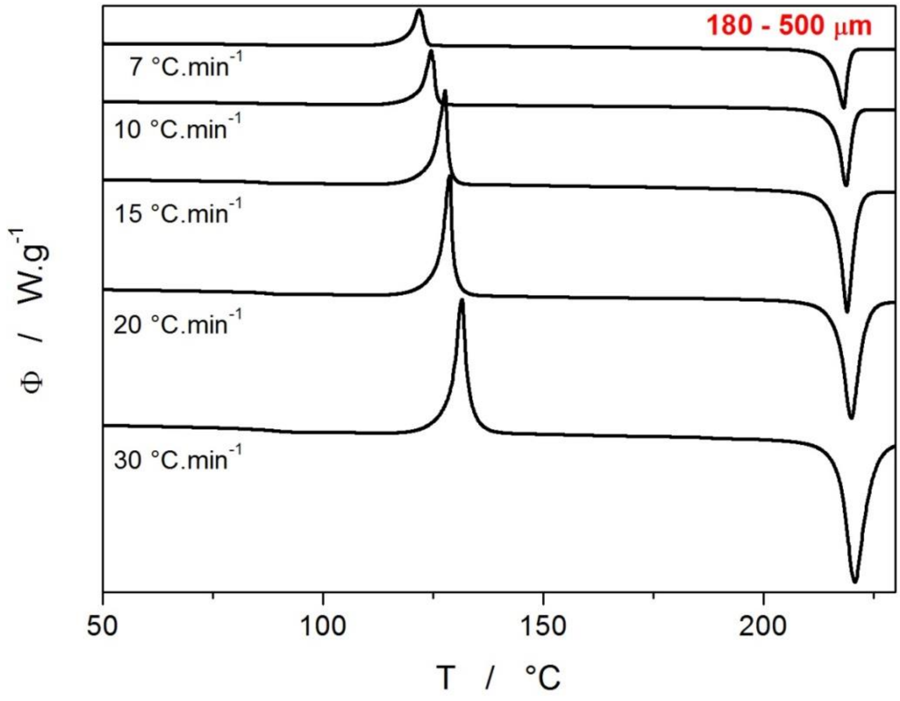

Figure S4: Zoomed-out data from Figure 1.

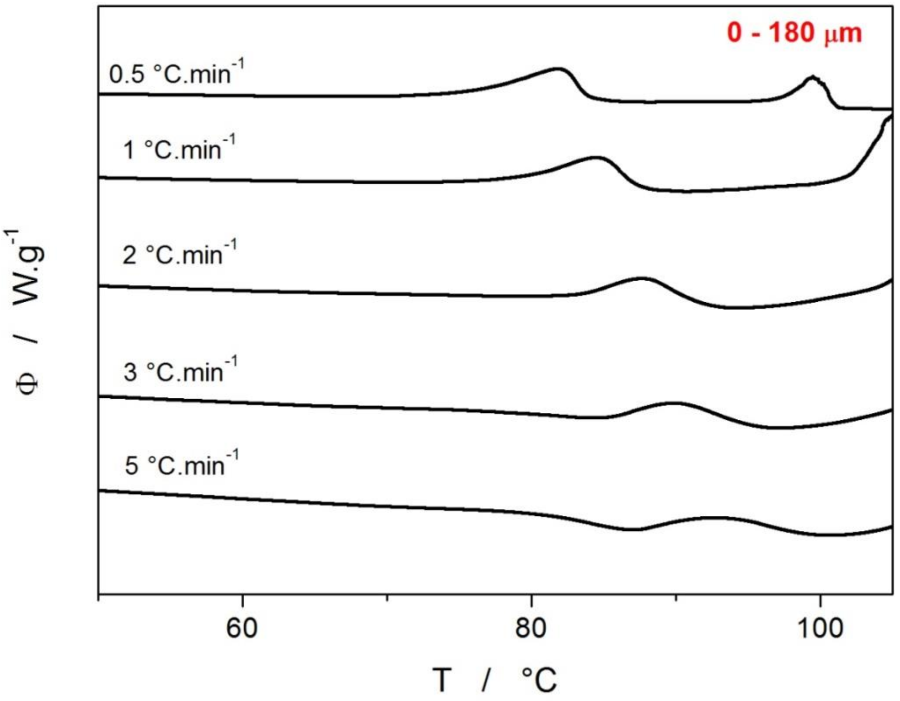

Figure S5: Zoomed-in data from Figure 1.

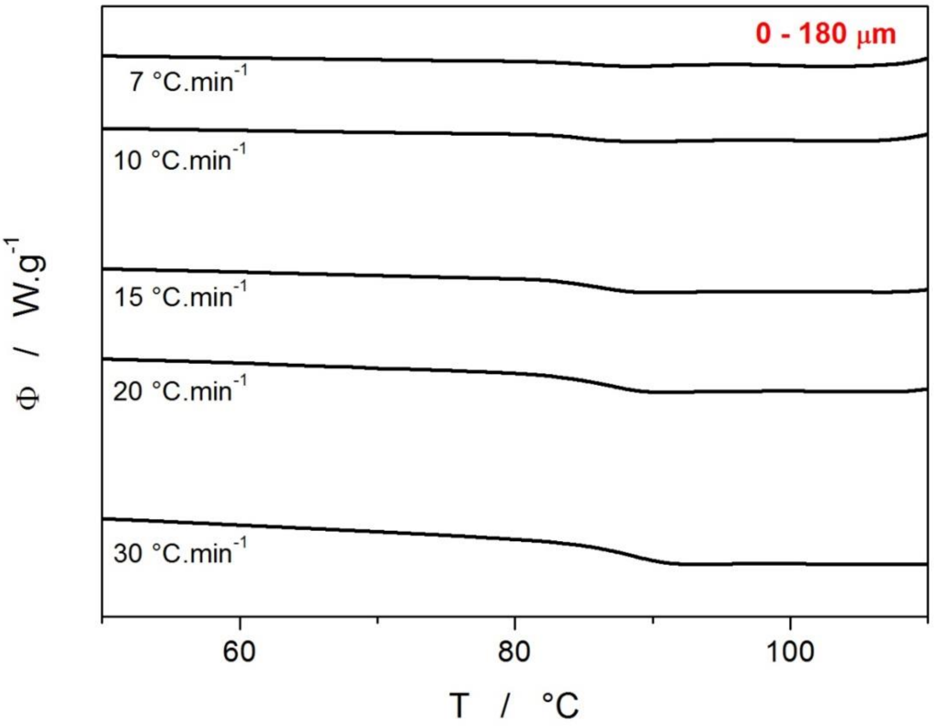

Figure S6: Zoomed-in data from Figure 1.

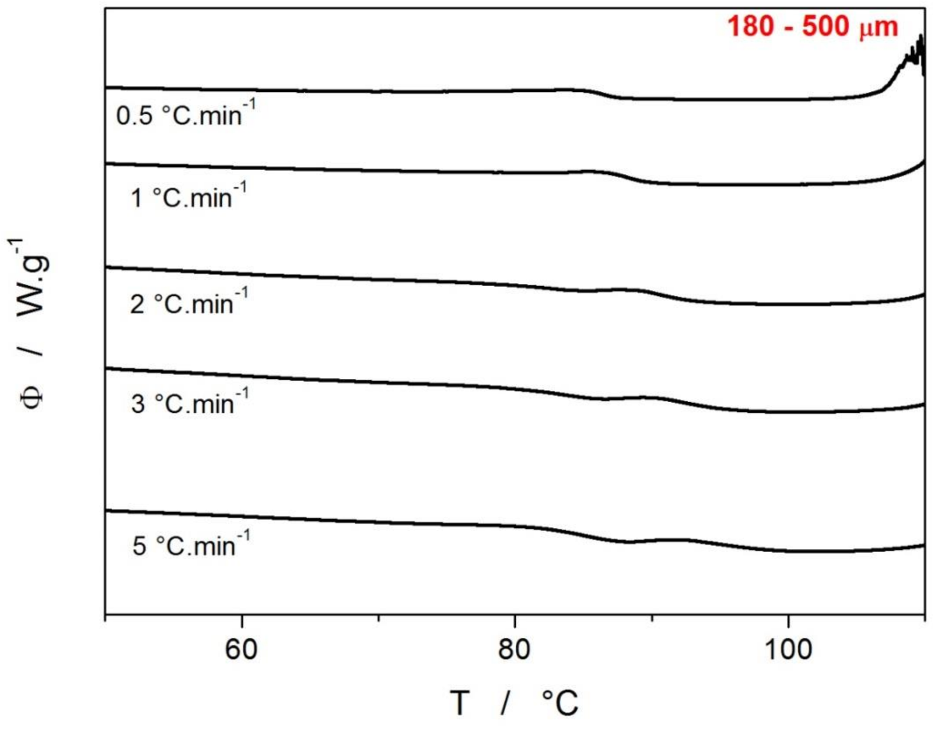

Figure S7: Zoomed-in data from Figure 1.

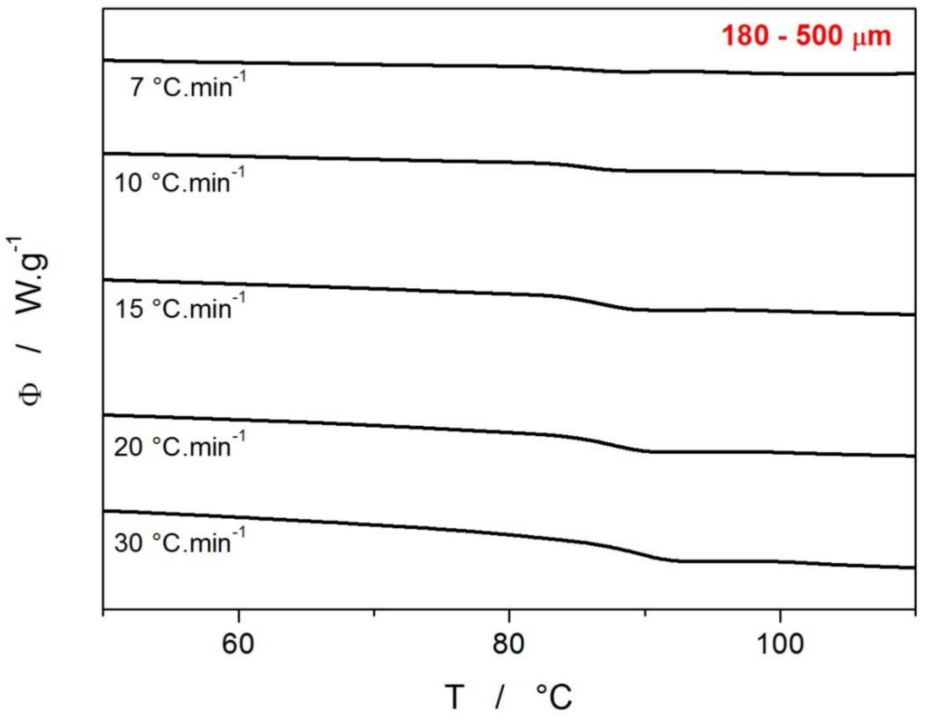

Figure S8: Zoomed-in data from Figure 1.

Part 2

Base characterization of DSC curves from Figure 1. ΔH_{c1} and T_{p1} denote crystallization enthalpy and temperature corresponding to the maximum of the peak for the crystallization pre-peak; similar quantities indexed „2“ denote the main crystallization peak. ΔH_m and T_m correspond to the melting enthalpy and extrapolated onset of the melting peak.

Table S1: Characteristic temperatures and enthalpies evaluated from the DSC curves

0 – 180 µm

q^+ / °C.min^{-1}	T_g / °C	ΔH_{c1} / J.g^{-1}	T_{p1} / °C	ΔH_{c2} / J.g^{-1}	T_{p2} / °C	ΔH_m / J.g^{-1}	T_m / °C
0.5		36.33	81.8	18.54	99.46	104.2	210.17
1		17.35	84.53	35.35	104.95	90.71	212.12
2	80.4	6.6	87.77	52.26	110.06	106	213.09
3	81.57	3.7	90.06	58.51	112.87	119.1	214.06
5	82.84	1.97	92.92	60.81	115.72	121.9	214.81
7	84.41	1.02	96.13	67.75	118.26	123	215.24
10	84.54			68.04	119.79	120.1	215.52
15	85.45			74.94	122.85	124.3	216.08
20	85.69			77.97	123.95	126.4	216.14
30	87.5			72.39	127.35	115.6	216.98

180 – 500 µm

q^+ / °C.min^{-1}	T_g / °C	ΔH_{c1} / J.g^{-1}	T_{p1} / °C	ΔH_{c2} / J.g^{-1}	T_{p2} / °C	ΔH_m / J.g^{-1}	T_m / °C
0.5	79.92	9.94	84.07	40.33	109.69	111.7	211.09
1	81.1	2.46	86.26	55.92	112.36	105.7	213
2	82.6	1.38	88.87	62.22	117	114.2	214.57
3	83.16	0.94	90.22	66.8	118.85	118.9	214.91
5	84.07	0.6	92.44	65.16	120.56	117.1	215.41
7	84.3	0.14	93.9	68.89	121.77	116.1	215.07
10	84.6			68.94	124.53	111.1	216.07
15	85.4			83.06	127.66	131.8	216.1
20	85.49			79.62	128.79	120.6	216.33
30	86.25			73	131.56	112	216.88

Part 3

Parameters of the double-AC-process kinetic equation (Eq. 16 coupled with Eq. 17) enumerated by means of the sc-MKA method. Correlation coefficients for the individual fits are listed as well. The meaning of the individual quantities is as follows: A is a pre-exponential factor, E is the activation energy of crystallization, M and N are the kinetic exponents of the AC model, ΔH is the crystallization enthalpy, indices 1 and 2 correspond to the overlapping crystallization sub-processes that form the overall crystallization signal.

Table S2: Parameters of the standard kinetic equation for crystallization data.

0 - 180 µm ; crystallization pre-peak

q^+	$\log(A_1/s)$	E_1	N_1	M_1	$\log(A_2/s)$	E_2	N_2	M_2	$\Delta H_1/\Delta H$	ΔH	r^2
°C.min^{-1}	-	kJ.mol^{-1}	-	-	-	kJ.mol^{-1}	-	-	-	J·g^{-1}	-
0.5	28.64	209.3	0.59	0.79	28.40	209.3	0.81	0.42	0.28	34.98	0.9999
1	28.70	209.3	0.72	0.79	28.44	209.3	0.80	0.49	0.25	15.74	0.9999
2	28.62	209.3	1.15	0.68	18.21	209.3	9.00	0.90	0.57	10.98	0.9988
3	28.50	209.3	1.17	0.57	-1.88	209.3	9.00	0.90	0.49	10.05	0.9969
5	28.40	209.3	1.12	0.45	-2.02	209.3	9.00	0.90	0.37	7.67	0.9939
7	28.30	209.3	1.11	0.39	-0.21	209.3	9.01	0.92	0.33	6.76	0.9927

0 - 180 µm ; main crystallization peak

q^+	$\log(A_1/s)$	E_1	N_1	M_1	$\log(A_2/s)$	E_2	N_2	M_2	$\Delta H_1/\Delta H$	ΔH	r^2
°C.min^{-1}	-	kJ.mol^{-1}	-	-	-	kJ.mol^{-1}	-	-	-	J·g^{-1}	-
0.5	23.26	180.4	0.67	0.90	22.97	180.4	0.51	0.36	0.77	19.34	0.9990
1	23.15	180.4	0.73	0.88	22.84	180.4	0.51	0.31	0.71	36.22	0.9966
2	23.05	180.4	0.69	0.84	22.49	180.4	0.27	0.22	0.75	48.68	0.9992
3	23.05	180.4	0.78	0.84	22.51	180.4	0.31	0.38	0.59	58.54	0.9993
5	23.00	180.4	0.73	0.81	22.69	180.4	0.50	0.52	0.63	58.92	0.9999
7	22.95	180.4	0.65	0.78	22.67	180.4	0.62	0.49	0.68	65.91	0.9999
10	23.03	180.4	0.63	0.79	22.71	180.4	0.62	0.49	0.63	65.44	0.9998
15	23.12	180.4	0.62	0.86	22.85	180.4	0.77	0.61	0.40	72.44	0.9998
20	23.20	180.4	0.69	0.87	22.88	180.4	0.83	0.60	0.44	76.86	0.9997
30	23.22	180.4	0.80	0.91	22.86	180.4	0.99	0.63	0.33	70.13	0.9998

180 - 500 µm; crystallization pre-peak

q^+	$\log(A_1/s)$	E_1	N_1	M_1	$\log(A_2/s)$	E_2	N_2	M_2	$\Delta H_1/\Delta H$	ΔH	r^2
°C.min^{-1}	-	kJ.mol^{-1}	-	-	-	kJ.mol^{-1}	-	-	-	J·g^{-1}	-
0.5	45.68	323.8	1.78	0.48	44.92	323.8	0.76	0.46	0.17	6.13	0.9992
1	37.38	323.8	3.78	1.60	45.09	323.8	1.06	0.55	0.08	2.86	0.9976
2	45.63	323.8	1.68	0.74	45.05	323.8	1.24	0.56	0.12	1.35	0.9994
3	45.26	323.8	1.37	0.56	44.92	323.8	1.24	0.51	0.39	1.04	0.9995
5	44.74	323.8	1.02	0.47	45.15	323.8	1.33	0.52	0.40	0.43	0.9992

180 - 500 µm ; main crystallization peak

q^+	$\log(A_1/s)$	E_1	N_1	M_1	$\log(A_2/s)$	E_2	N_2	M_2	$\Delta H_1/\Delta H$	ΔH	r^2
°C.min^{-1}	-	kJ.mol^{-1}	-	-	-	kJ.mol^{-1}	-	-	-	J·g^{-1}	-
0.5	30.04	233.8	1.10	0.93	29.67	233.8	0.18	0.60	0.83	35.38	0.9551
1	30.05	233.8	1.08	0.94	29.55	233.8	0.14	0.53	0.74	48.99	0.9872
2	30.16	233.8	0.83	1.03	29.70	233.8	0.26	0.72	0.36	56.82	0.9924
3	30.24	233.8	0.67	1.06	29.76	233.8	0.41	0.73	0.31	60.82	0.9973
5	30.06	233.8	0.87	0.92	29.92	233.8	0.71	0.65	0.78	62.49	0.9976
7	30.17	233.8	0.86	0.95	29.73	233.8	0.76	0.56	0.51	67.02	0.9997
10	30.09	233.8	0.66	0.94	29.60	233.8	0.67	0.51	0.47	66.82	0.9994
15	30.09	233.8	0.59	0.97	29.57	233.8	0.69	0.57	0.33	80.37	0.9979
20	30.20	233.8	0.68	1.01	29.62	233.8	0.80	0.59	0.34	76.98	0.9979
30	30.14	233.8	0.98	0.99	29.54	233.8	1.04	0.56	0.37	70.17	0.9995
